# Genome-wide identification of the GRF family in sweet orange (*Citrus sinensis*) and functional analysis of the *CsGRF04* in response to multiple abiotic stresses

**DOI:** 10.1186/s12864-023-09952-8

**Published:** 2024-01-06

**Authors:** Ming-Kang Fu, Ying-Na He, Xiao-Yue Yang, Xi Tang, Min Wang, Wen-Shan Dai

**Affiliations:** College of Life Sciences, Gannan Normal University, National Navel Orange Engineering Research Center, Ganzhou, 341000 Jiangxi China

**Keywords:** *Citrus sinensis*, GRF transcription factor, Genome-wide identification, Expression pattern, Functional identification

## Abstract

**Background:**

Citrus is one of the most valuable fruits worldwide and an economic pillar industry in southern China. Nevertheless, it frequently suffers from undesirable environmental stresses during the growth cycle, which severely restricts the growth, development and yield of citrus. In plants, the growth-regulating factor (GRF) family of transcription factors (TF) is extensively distributed and plays an vital part in plant growth and development, hormone response, as well as stress adaptation. However, the systematic identification and functional analysis of GRF TFs in citrus have not been reported.

**Results:**

Here, a genome-wide identification of GRF TFs was performed in *Citrus sinensis*, 9 members of *CsGRFs* were systematically identified and discovered to be scattered throughout 5 chromosomes. Subsequently, physical and chemical properties, phylogenetic relationships, structural characteristics, gene duplication events, collinearity and *cis*-elements of promoter were elaborately analyzed. In particular, the expression patterns of the *CsGRF* genes in response to multiple phytohormone and abiotic stress treatments were investigated. Predicated on this result, *CsGRF04*, which exhibited the most differential expression pattern under multiple phytohormone and abiotic stress treatments was screened out. Virus-induced gene silencing (VIGS) technology was utilized to obtain gene silenced plants for *CsGRF04* successfully. After the three stress treatments of high salinity, low temperature and drought, the *CsGRF04*-VIGS lines showed significantly reduced resistance to high salinity and low temperature stresses, but extremely increased resistance to drought stress.

**Conclusions:**

Taken together, our findings systematically analyzed the genomic characterization of *GRF* family in *Citrus sinensis*, and excavated a *CsGRF04* with potential functions under multiple abiotic stresses. Our study lay a foundation for further study on the function of *CsGRFs* in abiotic stress and hormone signaling response.

**Supplementary Information:**

The online version contains supplementary material available at 10.1186/s12864-023-09952-8.

## Background

Citrus is one of the most popular fruits worldwide. With a documented history of citrus cultivation spanning more than 4000 years, China acts as an influential hub of citrus origin in the globe [1]. At present, China leads the world in both citrus production and area. In recent years, the citrus industry in China has been developing rapidly, and has become a major pillar industry of the rural economy in the southern main production areas. Further positive contributions have been made to promoting income of farmers, expanding employment for urban and rural residents and improving the ecological environment [2]. Sweet orange (*Citrus sinensis*) belongs to one of the largest proportion and most economically valuable species in *Citrus*. It abounds in vitamin C, citric acid, dietary fiber and pectin, which functions in cosmetology, fatigue elimination and laxation [3]. Meanwhile, studies have proved that sweet orange could lower cholesterol and blood pressure, expand the coronary artery of the heart, and is a healthy fruit to prevent coronary heart disease and atherosclerosis [4]. Collectively, sweet orange has become one of the favorite fruits by virtue of its delicious and juicy taste and nutritious value. However, sweet orange, as the most widely cultivated and economically efficient species in the Citrus, has relatively low adaptability to the environment, and often suffers from a variety of adverse stresses, such as low temperature, drought, salinity, pests and diseases, which seriously restricts the improvement of the sweet orange industry [5]. Therefore, studies of the intrinsic response mechanism under adversity stress in sweet orange is one of the focuses of citrus science and technology in China.

Growth-regulating factors (GRFs) are an unique class of plant-specific transcription factors involved in regulating the growth and development of plant cotyledons, leaves, stems, roots, flowers, and seeds, as well as biotic and abiotic stress response processes. The first identified growth-regulatory factor was *OsGRF1* in rice [6], and subsequently the GRF family was gradually recognized in various plants, such Arabidopsis, rice, tomato, tobacco, poplar and wheat [7–12]. These findings indicate that *GRF* genes are mainly expressed in the meristem of plants and plays an essential role in plant growth and development. Generally, the members of GRFs in terrestrial plants ranges from 8–20, for instance, 9 in Arabidopsis, 12 in rice, 14 in maize, and 17 in Chinese cabbage [7, 8, 13]. Typical GRF proteins contain one conserved QLQ (glutamine-leucine-glutamine, Glu-Leu-Glu) damain and one or two conserved WRC (tryptophan-arginine-cysteine, Trp-Arg-Cys) domains at the N-terminus [14]. Studies have shown that the QLQ domain exists in all Eukaryote genomes and serves as an essential protein-interacting region through which GRFs exercise transcriptional activation by interacting with the SNH domain of the GRF-interacting factor (GIF) [15]. The WRC domain is unique to plants and contains a DNA binding domain and a nuclear localization signal domain. It is crucial for guiding GRF protein into the nucleus and binding to *cis*-acting elements of their target genes to regulate the expression of downstream genes [16].

A complex relationship exists between GRFs and phytohormones and varies greatly among species. Numerous hormone-related *cis*-acting elements were identified in the promoter region of *SitGRFs* in foxtail millet, mainly including *cis*-acting element involved in abscisic acid responsiveness (ABRE), MeJA-responsiveness (CGTCA/TGACG-motif), gibberellin-responsiveness (P-box), salicylic acid responsiveness (TCA-element) and auxin-responsiveness (TGA-element), implying that the GRF family may play a role in hormone response in foxtail millet [17]. Gibberellin (GA) regulates diverse aspects of plant growth and development [18]. Previous studies have shown that GA_3_ treatment induces up-regulation of *OsGRF1*/*2*/*3*/*7*/*10*/*12* and represses the expression of *OsGRF9* in rice, but results in reduced expression of most *GRFs* in cabbage [8, 13]. In addition, the effects of phytohormone including brassinolide (BR) and cytokinin (CK) on expression of *GRF* genes have also been reported. *MtGRF5* in *Medicago truncatula* was significantly down-regulated by BR treatment [19]. *AtGRF5* and cytokinins synergistically enhance cell division and chlorophyll retention after dark-induced senescence [20]. Furthermore, plenty of studies have established that GRFs play an imperative role in the response of plants to adverse external stresses. AtGRF7 protein from Arabidopsis binds to and represses *DREB2A*, an vital *cis*-acting element of the dehydration response, and functions as a repress regulator of a range of osmotic stress-responsive genes to maintain normal plant growth [21]. Suppression of *NtGRF7* in tobacco resulted in increased osmotic stress resistance, while down-regulation of *NtGRF1* and *NtGRF3* caused increased susceptibility of tobacco to *Phytophthora nicotianae* [22]. Taken together, GRFs function critically in phytohormone response and stress adaptation, but exactly which genes and how they respond varies considerably among species.

In this study, a comprehensively identification of the GRF family was performed in *C. sinensis*, a representative species of citrus. Expression patterns of *CsGRFs* under multiple phytohormone and abiotic stress treatments were analyzed. *CsGRF04*, which displayed extremely strong responsive to multiple phytohormones and abiotic stresses was screened and excavated. Virus-induced Gene Silencing (VIGS) were exploited to further validate the function of *CsGRF04* under abiotic stresses. This study provides resources for the genetic improvement and breeding of sweet orange, as well as a theoretical basis for elucidating the responsive mechanism of the GRF family under phytohormones and abiotic stresses in *C. sinensis*.

## Materials and methods

### Plant cultivation and multiple stress treatments

Two-month-old seedlings of sweet orange (*C*. *sinensis*), grown in a greenhouse of National Navel Orange Engineering Research Center, Gannan Normal University, were used to analyze the expression of *CsGRFs*. Plants were kept in growth chambers under 16: 8 h, light: dark conditions at a temperature of 25 °C. For the phytohormone treatments, plants were irrigated and foliar sprayed with 100 mL of 100 mM abscisic acid (ABA), 500 μM salicylic acid (SA), 200 μM jasmonic acid (JA), 5 mg/L gibberellin (GA) and 20 mg/L ethrel (ETH), respectively [23–27]. For cold treatment, seedlings of sweet orange were placed in a incubator set at 4 °C for 0 h, 3 h, 6 h, 12 h, 24 h and 48 h. For dehydration treatment, seedlings were airdried on filter papers at ambient temperature for 0.5 h, 1 h, 3 h, 6 h and 12 h. For salt treatment, each potted sweet orange seedlings were sprayed with 100 mL 300 mM NaCl solution for 0 h, 3 h, 6 h, 12 h, 24 h and 48 h [24, 25]. Leaves were randomly collected at designated time points, which was 0 h, 3 h, 6 h, 12 h, 24 h and 48 h after treatments for RNA extraction, with three biological replicates for each experiment. All samples were instantly frozen in liquid nitrogen and stored in a refrigerator at -80 °C for gene expression analysis.

### Identification of the GRF genes in *C. sinensis*

The Hidden Markov Models (HMM) of the conserved domains QLQ (PF08880) and WRC (PF08879) were downloaded from the Pfam protein family database (http://pfam.xfam.org/), and the HMMER search program (http://hmmer.janelia.org/, Version 3.0) was conducted using the two HMM files as query sequences to identify putative GRF proteins employing BLASTp search against the *C. sinensis* genome (http://citrus.hzau.edu.cn/index.php) (E-value ≤ 1e^−5^) [28]. The putative GRF proteins were further submitted to SMART (http://smart.embl-heidelberg.de/) and CDD (http://www.ncbi.nlm.nih.gov/Structure/cdd/wrpsb.cgi) online website to confirm the presence of the complete QLQ and WRC domains [29, 30]. All non-redundant protein with longest transcript sequences were retained after eliminating the sequences harboring incomplete conserved domains. The relative molecular weight (MW) and isoelectric point (pI) of the ascertained CsGRF proteins were calculated using the Calculate pI/MW tool ExPASy (https://web.expasy.org/compute_pi/) [31].

### Phylogenetic, gene structure and conserved motif analysis

The GRF protein sequences of Arabidopsis (*Arabidopsis thaliana*), rice (*Oryza sativa subsp. japonica*), poplar (*Populus trichocarpa*), pear (*Pyrus bretschneideri*) and grape (*Vitis vinifera*) were downloaded from the ensembl website (http://asia.ensembl.org/index.html) [32]. Together with GRF proteins of *C. sinensis*, a multiple sequence alignment of GRF proteins from these six species was performed through the ClustalW (https://www.genome.jp/tools-bin/clustalw, Version 2.0) [33]. A MEGA (https://www.megasoftware.net/, Version 11.0) software was employed to construct the phylogenetic analysis of GRF proteins based on amino acid sequences by using the neighbor-joining method with the maximum likelihood method (bootstrap: 1,000 replicates) [34]. TBtools software (Version 1.120) was employed to illustrate the gene structure based on the genomic GFF file of *C*. *sinensis* [35]. Conserved motifs of CsGRFs were identified using MEME program (http://meme-suite.org/tools/meme, Version 5.4.1) with the following parameters: maximum number of motifs of 6 and the optimum width from 6–100 amino acid residues [36].

### Gene duplication and collinearity analysis

The accurate locations on the chromosomes for the genes encoding the CsGRF proteins were obtained from the Citrus Pan-genome to Breeding Database. All *CsGRF* genes were mapped separately onto the nine chromosomes in ascending order of physical position (bp), from the short-arm telomere to the long-arm telomere. Intraspecifc and interspecies synteny analyses were performed by the MCScanX software (http://chibba.pgml.uga.edu/mcscan2/) with the flowing parameters: match score (> 20); gap penalty (-1); match size (5); E-value: 1e^−5^; max gaps (25), and respectively visualized using the “Amazing Super Circo” and “Multiple Synteny Plot” modules of the TBtools software (Version 1.120) [35, 37].

### *cis*-acting elements analysis

The 2.5 kb upstream promoter sequences from the transcription start site of 9 *CsGRF* genes were extracted for *cis*-acting elements analysis by using the ‘Sequence Fetch’ tool of Citrus Pan-genome to Breeding Database. Two plant *cis*-elements online database, New PLACE (https://www.dna.affrc.go.jp/PLACE/?action=newplace) and PlantCARE (http://bioinformatics.psb.ugent.be/webtools/plantcare/html/), were used to analyze the stress-response elements in promoters of *CsGRFs* [38, 39]. The identified *cis*-acting elements were then visualized by TBtools (Version 1.098696) [35].

### RNA extraction and quantitative real-time PCR (qRT-PCR) analysis

According to the manufacturer’s instructions, total RNA was prepared using MiniBEST Universal RNA Extraction Kit (Takara, Japan) from leaves collected at designated time points. Agarose gel electrophoresis and NanoDrop 2000 spectrophotometer (Thermo, USA) were employed to verify the quality and integrity of total RNA. The first-strand cDNA was reverse-transcribed from RNA by PrimeScript™ RT reagent Kit with gDNA Eraser (Takara, Japan). The specific primers of *CsGRF* genes were designed using “Batch qPCR Primer Design” modules of the TBtools software (Version 1.120) [35] and listed in Supplementary Table S1. Realtime qRT-PCR analysis was done using SYBR GREEN PCR Master Mix (TaKaRa, Japan) on a QuantStudio 5 Applied BioSystem (ThermoFisher Scientific, USA) to investigate the expression of *CsGRFs* after treatments with multiple phytohormones and abiotic stresses in *C. sinensis*. The *CsActin* gene was used as an internal reference, each reaction was repeated in three biological and technical replicates, and the 2^−ΔΔCt^ method was applied to calculate the relative expression levels. Heatmaps were established using TBtools (Version 1.098696) based on transformed log_2_ values. Venn diagram were generated using “Venn and Upset Plot” modules of the TBtools software (Version 1.120) to depict number of treatments commonly shared by up- and down-regulated genes after multiple treatments, respectively.

### Generation of *CsGRF4*-silenced plants

To obtain VIGS-mediated gene suppressing plants, 350 bp fragments of *CsGRF4* were amplified and inserted into *BamH* I and *Sma* I sites of pTRV2 vector (Tobacco Rattle Virus-based 2). The pTRV1 (empty vector) and fusion constructs (pTRV2-*CsGRF4*) were separately transformed into *A. tumefaciens* strain GV3101 by heat shock. The bacterial suspensions of pTRV1 were co-transformed with the recombinants in a 1: 1 volume ratio in 2-(Nmorpholino) ethanesulfonic acid (MES) buffer (10 mM MgCl_2_, 10 mM MES, and 200 mM acetosyringone, pH 5.6) and kept in dark for at least 2 h at room temperature as described previously [24, 25]. The germinated *C. sinensis* seeds (about 1–2 cm) were immersed in the bacterial mixtures and placed in a vacuum chamber. After 10 min vacuum infiltration at 0.8–0.9 MPa, the transformed seeds were cultivated for 3 days at dark and then transplanted to soil pots. One month later, fully expanded leaves were collected from each plant and subjected to genomic PCR as well as qRT-PCR analysis for positive identification and silencing efficiency detection.

### DNA isolation and positive identification of *CsGRF04*-VIGS plants

Young leaves of WT and *CsGRF04*-VIGS plants were selected for genomic DNA isolation using the cetyltrimethylammonium bromide (CTAB) method. The specific primers used for positive identification were designed using Primer Premier 5.0 software (Supplementary Table S1). With the extracted genomic DNA as a template, the pTRV2 vector forward primer and *CsGRF04*-pTRV2 reverse primer were used for positive identification. Cirus DNA that could be successfully amplified with a PCR product of 592 bp fragments by the above primers were considered to be from positive plants which were retained for qRT-PCR to detect the transcript abundance.

### Abiotic stress tolerance assays

For salt stress tolerance assay, 1-month-old *CsGRF4*-VIGS and wild type (WT) potted plants were sprayed with 100 mL of 300 mM NaCl solutions at 3 days intervals for 2 weeks. For cold stress tolerance assay, 1-month-old *CsGRF4*-VIGS and WT plants were exposed to a mild stress treatment at 4 °C for 24 h, and then exposed to a severe cold stress at -4 °C for 8 h, followed by a recovery period at ambient temperature for 3 days. For drought tolerance assay, 1-month-old *CsGRF4*-VIGS and WT plants were grown for 1 week under a full watering regime, followed by deprivation of watering for 3 weeks. Leaves from each assay were randomly sampled before and after treatments for physiological analysis. Electrolyte leakage (EL) was measured by investigating relative conductance as described by prior method [5], and chlorophyll content was extracted and analyzed according to prior study [40].

### Statistical analysis

Stress treatments were repeated at least three times independently. Data were evaluated by Tukey’s multiple test in ANOVA program of SAS software package (SAS Institute, Cary, NC, USA). Statistical significance were considered at *p* < 0.05.

## Results

### Identification and phylogenetic analysis of the *CsGRF* family

To identify GRF family genes in *C*. *sinensis*, HMM profile from the Pfam database and BLASTp search were performed against reference genomes using the consensus sequence of QLQ and WRC domain, respectively. Initially, A total of 19 *CsGRF* genes were identified, and 9 *CsGRF* genes were retained after eliminating the redundant sequences and sequences containing only partial QLQ or WRC domains. Detailed information of *CsGRFs* were presented in Supplementary Table S1. According to their chromosomal positions, the identified *CsGRFs* were named as *CsGRF01*-*CsGRF09*, ranging from 232 aa (*CsGRF04*) to 600 aa (*CsGRF02*) in length, and the coding sequences (CDSs) of *CsGRFs* ranged from 699 bp (*CsGRF04*) to 1803 bp (*CsGRF02*) in length. Additionally, the molecular weights ranged from 25.26 kDa (*CsGRF08*) to 65.05 kDa (*CsGRF03*), and the isoelectric points were between 5.48 (*CsGRF01*) and 9.75 (*CsGRF08*). The pI values of 8 CsGRF members except CsGRF01 was greater than 7, indicating that most of the CsGRF proteins are enriched with basic amino acids (Supplementary Table S2).

**Fig. 1:**
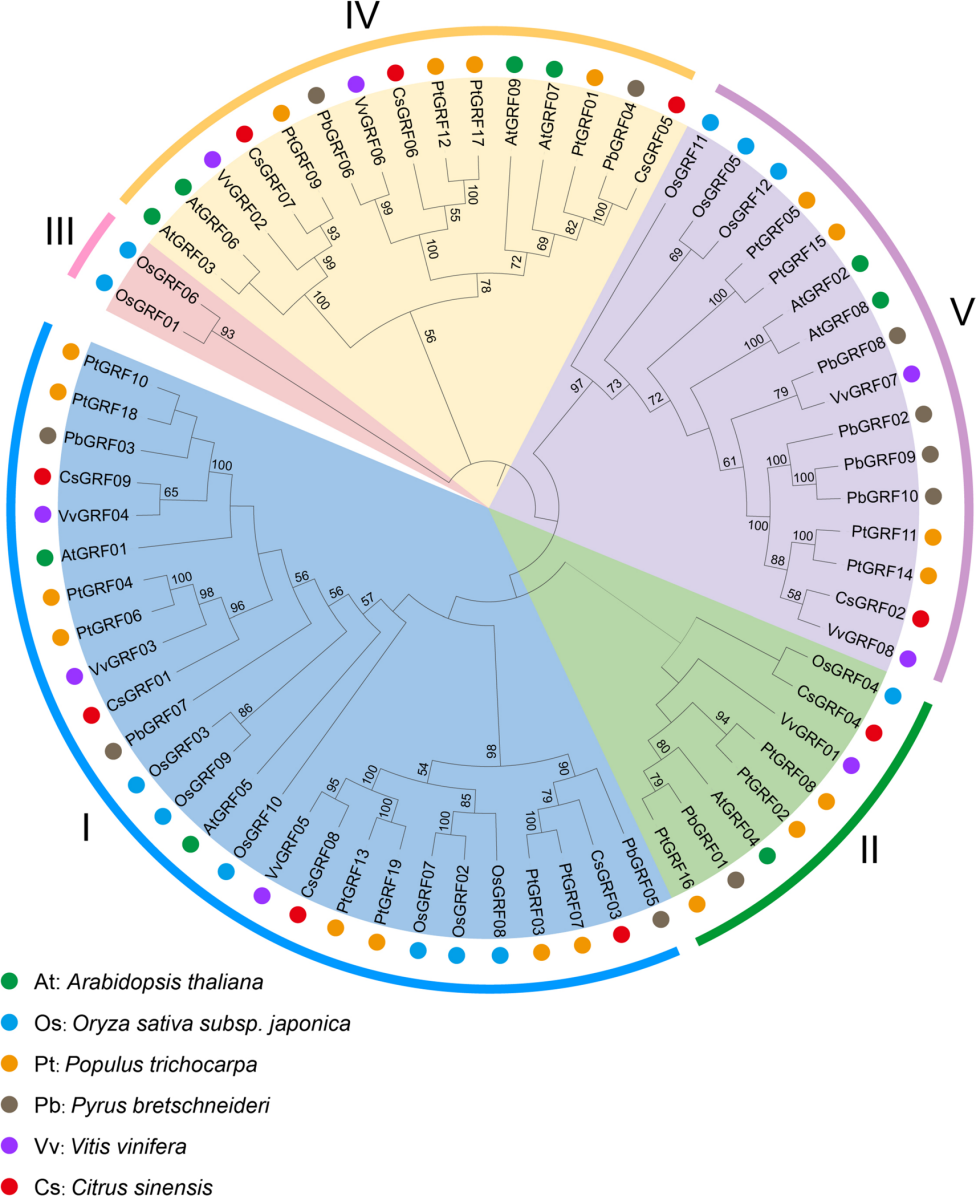
Phylogenetic analysis of GRFs in *Citrus sinensis* (Cs), *Arabidopsis thaliana* (At), *Oryza sativa subsp. japonica* (Os), *Populus trichocarpa* (Ptr), *Pyrus bretschneideri* (Pb) and *Vitis vinifera* (Vv). The phylogenetic tree was created using MEGA X by the Neighbor-Joining (NJ) method with 1,000 bootstrap replicates. The species background for each GRF protein is represented by different colors. Based on the bootstrap values and evolutionary distances, the tree was clustered into five subfamilies (I-V)

To gain insight into the evolution of GRFs in *C. sinensis*, a neighbor-joining phylogenetic tree was constructed using the GRF proteins from representative plant species, including *Arabidopsis thaliana* (At, 9 members), *Oryza sativa subsp. japonica* (Os, 12 members), *Populus trichocarpa* (Ptr, 19 members), *Pyrus bretschneideri* (Pb, 10 members) *Vitis vinifera* (Vv, 8 members) and *Citrus sinensis* (Cs, 9 members) (Fig. 1). The phylogenetic analysis revealed that the 67 GRF proteins from six species were divided into five major clusters, designated as clusters I to V, and the distribution of the 9 *CsGRF* genes among the clusters were not even. Among the five clusters, cluster I is relatively considerable and contains 4 members, namely *CsGRF01*, *CsGRF03*, *CsGRF08* and *CsGRF09*. Followed by cluster IV, which includes *CsGRF05*, *CsGRF06* and *CsGRF07* with a total of 3 members. Cluster II and V contain the least number of *CsGRFs*, both with only one member (*CsGRF04* and *CsGRF02*, respectively). Noteworthily, no cluster III *GRF* members could be found in *C*. *sinensis*. Compared with monocotyledons, most *CsGRF* members displayed more closely relationship to the dicotyledons like *V*. *vinifera*, *P. bretschneideri* and *P*. *trichocarpa.* For instance, *CsGRF08* and *VvGRF05* from cluster I, *CsGRF02* and *VvGRF08* from cluster V, *CsGRF05* and *PbGRF04* along with *CsGRF07* and *PtGRF09* from cluster IV, all of which gene pairs shared a high sequence similarity between each other (Fig. 1).

### Conserved domain and gene structure analysis of *CsGRFs*

**Fig. 2:**
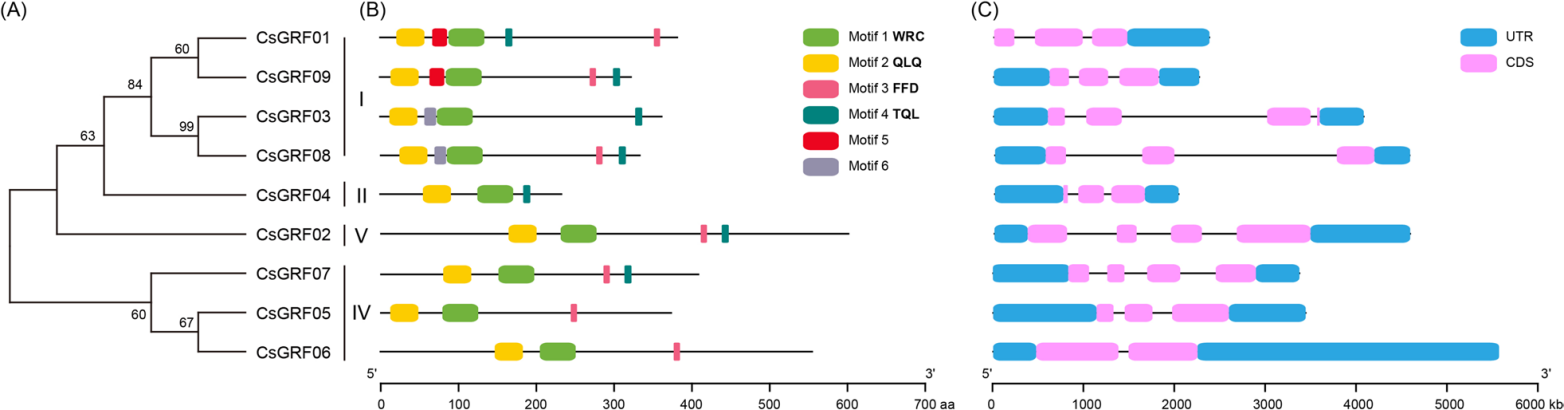
Conserved motif and gene structure analysis of *CsGRFs.* **A** Phylogenetic relationship of CsGRF proteins. **B** The distribution of 6 conserved motifs in CsGRF proteins, identified by MEME program, was shown by different colored blocks. The sequences of these conserved motifs were listed in Supplementary Table S3. **C** Exon/intron structures of *CsGRFs*. The exons and introns were represented by pink boxes and black lines, respectively. The blue boxes indicated the upstream and/or downstream untranslated region

GRF proteins usually possess two conserved motifs of QLQ and WRC that might be involved in activating the functions of GRF proteins [41]. Meanwhile, other motifs may also serve unknown functional or structural roles along with the QLQ and WRC domain. To further investigate the structural diversity and functional prediction of the *CsGRF* genes, we firstly analyzed the conserved domain of CsGRF. Totally, 10 conserved motifs were identified and the length of these motifs ranged from 9 to 47 amino acids (Supplementary Table S3). Among them, motif 1 and 2 were respectively annotated as the WRC and QLQ domain, and were possessed by all CsGRF family members. All family members except CsGRF05 and CsGRF06 contained a TQL (Thr, Gln, Leu) domain at the C-terminus, while the C-terminals of all CsGRF members except CsGRF03 and CsGRF04 harbored an FFD (Phe, Phe, Asp) domain (Fig. 2A, B). A multiple sequence alignment of the core QLQ and WRC domain of CsGRFs was shown in Supplementary Figure S1. It is worth noting that the features of these motifs were conservative among same clusters, for instance, all 3 CsGRFs from group IV (CsGRF05-CsGRF07) contained three common conserved motifs (motif 1, 2 and 3). To further investigate the structural diversity of the *CsGRF* genes, we analyzed the distribution of introns/exons by comparing genomic and CDS sequences. The results revealed that most *CsGRF* genes harbored 3–4 exons, with the exceptions being *CsGRF06*, which have 2 exons. Furthermore, the position and structure of introns/exons were commonly well-conserved in *CsGRFs* from same clusters. For example, *CsGRF01*, *CsGRF08* and *CsGRF09* from cluster I contain 3 exons at similar position (Fig. 2C).

### Duplication and synteny analysis of *CsGRF* gene members

**Fig. 3:**
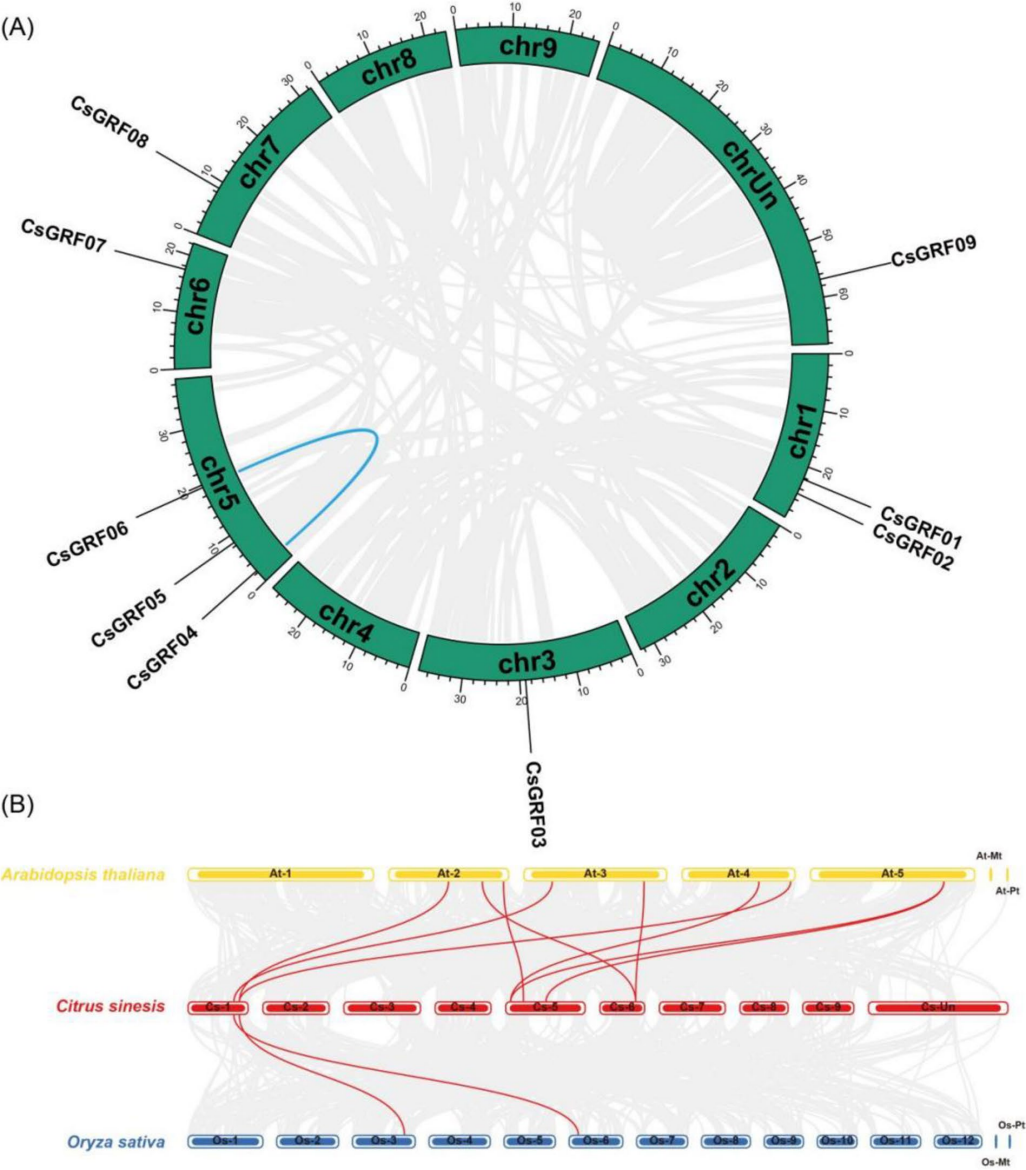
Duplication and synteny analyses of *GRF* genes among *Citrus sinensis, Arabidopsis thaliana* and *Oryza sativa*. **A** Location and the collinearity analysis of *CsGRFs*. The green columns represent chromosomes with the chromosome numbers placed in the middle and the gene ID shown outside the plot. The blue line inside the plot indicated the the genes located on the duplicated segmental regions between *CsGRFs*. **B** Collinearity relationship of *GRF* genes among *Citrus sinensis, Arabidopsis thaliana* and *Oryza sativa*. The horizontal columns represent chromosomes with the chromosome numbers placed in the middle. The gray lines indicated the collinear blocks within each two genome pairs, and the identified syntenic *CsGRF* genes are linked by red lines

The distribution characteristics of *CsGRFs* on chromosomes were extracted from the genome GFF annotation file of *C. sinensis*. The visualized results showed that the chromosomal distribution of *CsGRFs* was heterogeneous, with varying densities of gene distribution on different chromosomes. Most of the genes (3 *CsGRFs*) were located in chromosome 5, followed by chromosome 1 (2 *CsGRFs*). Chromosomes 3, 6, and 7 contained only one *CsGRF* gene. Only one gene, *CsGRF09*, could not be located in any definite chromosome (Fig. 3A).

It is well recognized that gene duplication events are instrumental in generating gene mutations in plants and thus differentiate the functions of ancestral genes that are critical for plant adaptation [42]. To further examine the evolution of *GRF* genes in *C. sinensis*, genome duplication events were investigated for segmental and tandem duplications. An intraspecifc collinearity analysis showed that only one pair of *CsGRFs* originated from segmental replication (*CsGRF04* and *CsGRF06*) on chromosome 5 in *C*. *sinensis* (represented by blue line in Fig. 3A). No tandem duplication events were detected among *CsGRF* genes in *C*. *sinensis* genome, suggesting that segmental duplication events dominated the expansion of *CsGRF* family.

To further characterize the evolution of *CsGRFs*, the dicotyledonous Arabidopsis (*Arabidopsis thaliana*) and monocotyledonous rice (*Oryza sativa subsp. japonica*) were selected as reference genomes, and the genomic collinearity of *CsGRF* with *AtGRF* and *OsGRF* was plotted. As shown in Fig. 3B, 9*CsGRF* genes were collinear with the *AtGRF* genes, and only 2 *CsGRF* genes were collinear with the *OsGRF* genes, indicating that the *CsGRF* gene family is more closely related to *A*. *thaliana* than *O*. *sativa*, which may be related to the fact that *C. sinensis* and Arabidopsis belong to the same group of dicotyledonous plants and have closer evolutionary relationships.

### *cis*‑element analysis of *CsGRF* promoters

**Fig. 4:**
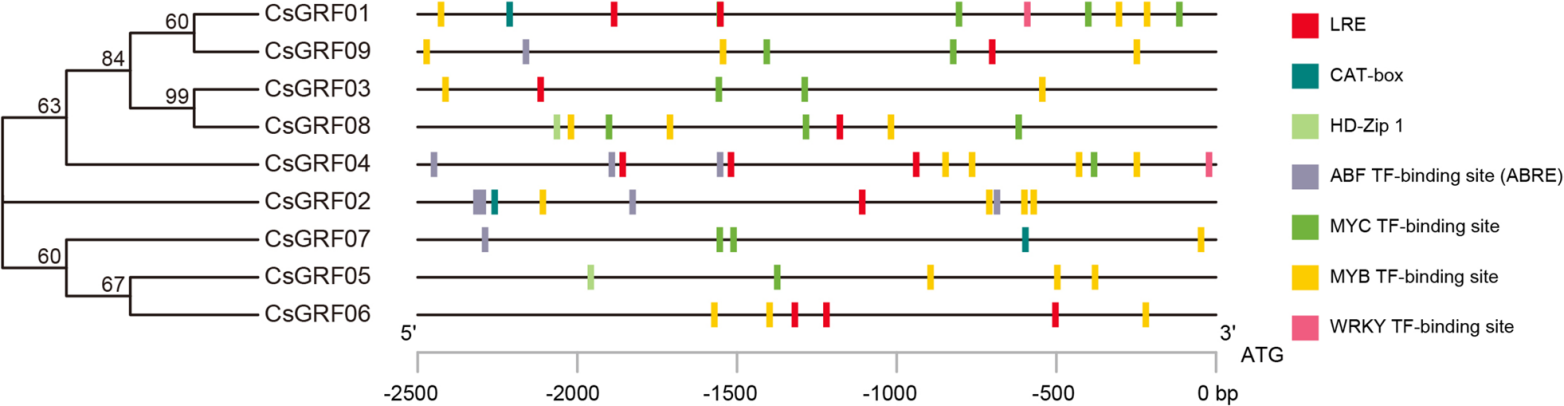
*cis*-element analysis in the promoters of *CsGRFs*. *cis*-regulatory stress-responsive elements were identified in the 2.5 kb upstream promoter region of *CsGRFs*. Different colored rectangles represent different elements. Detailed information of sequence and position of these elements was described in Supplementary Table S6

Promoter *cis*-elements play vital roles in the initiation of gene expression [43]. To better understand the potential functions and regulatory mechanisms of the *CsGRF* family genes, The 2.5 kb sequence upstream of each *CsGRF* were extracted for *cis*-element analysis. Besides to the core promoter elements TATA-box and CAAT-box (not shown in the figure due to the large quantity), numerous *cis*-acting elements related to phytohormone response, environmental stress, growth and developmental processes existed in the promoter region of *CsGRFs* (Fig. 4, Supplementary Table S4). For instance, the abscisic acid responsive element (ABRE, 9), the light response element (LRE, 12), the *cis*-acting regulatory element related to meristem expression (CAT-box, 3), and the *cis*-element involved in differentiation of the palisade mesophyll cells component (HD-Zip 1, 2). Furthermore, a large number of transcription factor (TF) binding sites existed on the promoters of *CsGRF* family genes, such as MYB-TF-binding sites (27), MYC-TF-binding sites (15), WRKY-TF-binding sites (2), and ABF-TF-binding sites (9), and it was hypothesized that the *CsGRF* family might be involved in the processes of phytohormone signaling, response to environmental stresses, and transcriptional regulation, with possible differences in the expression patterns of *CsGRF* family genes.

### Expression profles of *CsGRFs* under multiple phytohormone treatments

**Fig. 5:**
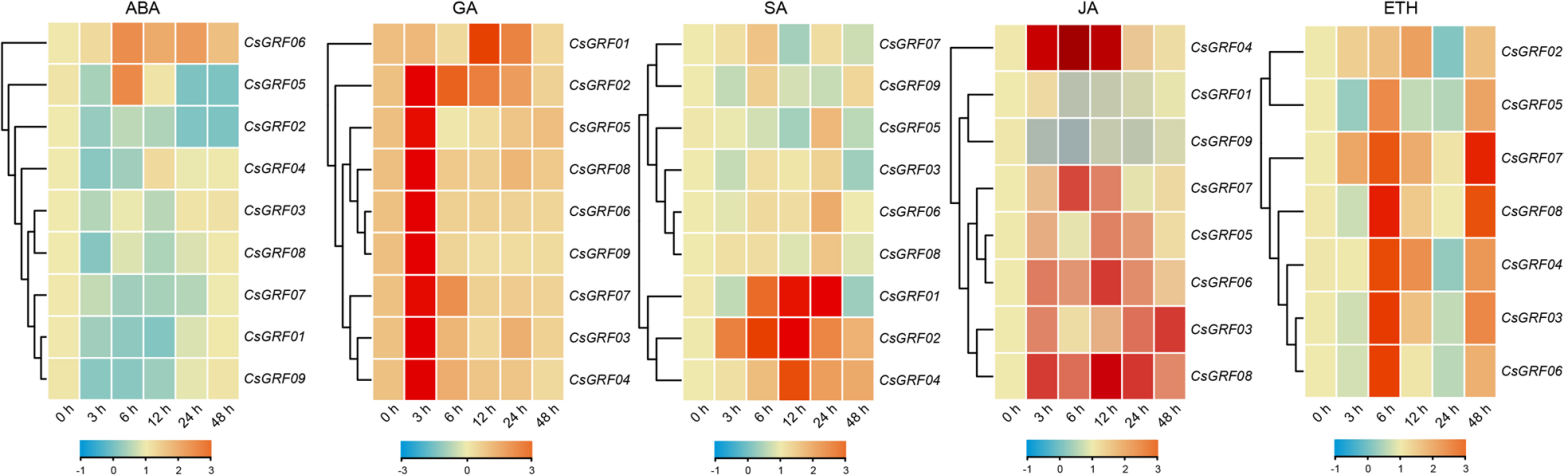
Expression profiles of *CsGRF*s under multiple phytohormone treatments. Expression analysis was carried out in leaves of *C*. *sinensis* at different time points (0 h, 3 h, 6 h, 12 h, 24 h and 48 h after treatments). The qPCR results of *CsGRFs* were normalized by log_2_ transform. The heatmap constructed by TBtools software. Color scale erected horizontally at the bottom of the diagram

Promoter analysis showed that a substantial number of *cis*-acting elements associated with phytohormone responses and abiotic stress enriched in the promoter region of the *CsGRFs*, suggesting its possible involvement in these biological processes. To gain insights into the potential functions of the *CsGRF* genes in response to phytohormones, qRT-PCR were performed to analyze the expression patterns of all *CsGRF* genes under five phytohormone treatments, which were ABA, GA, SA, JA, and ETH. All 5 phytohormone treatments induced up/down-regulation in the expression of multiple *CsGRF* genes with different degrees, but there were discrepancies in the way they responded. The vast majority of *CsGRFs* expression showed significant down-regulation under ABA treatment, with only *CsGRF06* exhibiting up-regulated pattern. All *CsGRFs* exhibited a significant up-regulation induction after GA treatment, with most *CsGRFs* reaching a peak level at 3 h after treatment, indicating that *CsGRFs* were highly and rapidly responsive to GA treatment. After SA treatment, the expression levels of 4 genes, *CsGRF01*, *CsGRF02*, *CsGRF04* and *CsGRF06*, showed an up-regulated induced pattern, while *CsGRF05* exhibited a down-regulated induced pattern, and the rest of the *CsGRFs* displayed no remarkable differences in transcript abundance. Expression levels of *CsGRF02* could not be detected under JA treatment, most of the *CsGRFs* showed extremely up-regulated expression patterns under JA treatment, except for *CsGRF01* and *CsGRF09* which exhibited down-regulation expression levels. Similar situation occurred after ETH treatment, where all gene expressions showed up-regulation induction, except for *CsGRF01* and *CsGRF09*, which were undetectable (Fig. 5, Supplementary Table S5).

### Expression profles of *CsGRFs* under multiple abiotic stresses

**Fig. 6:**
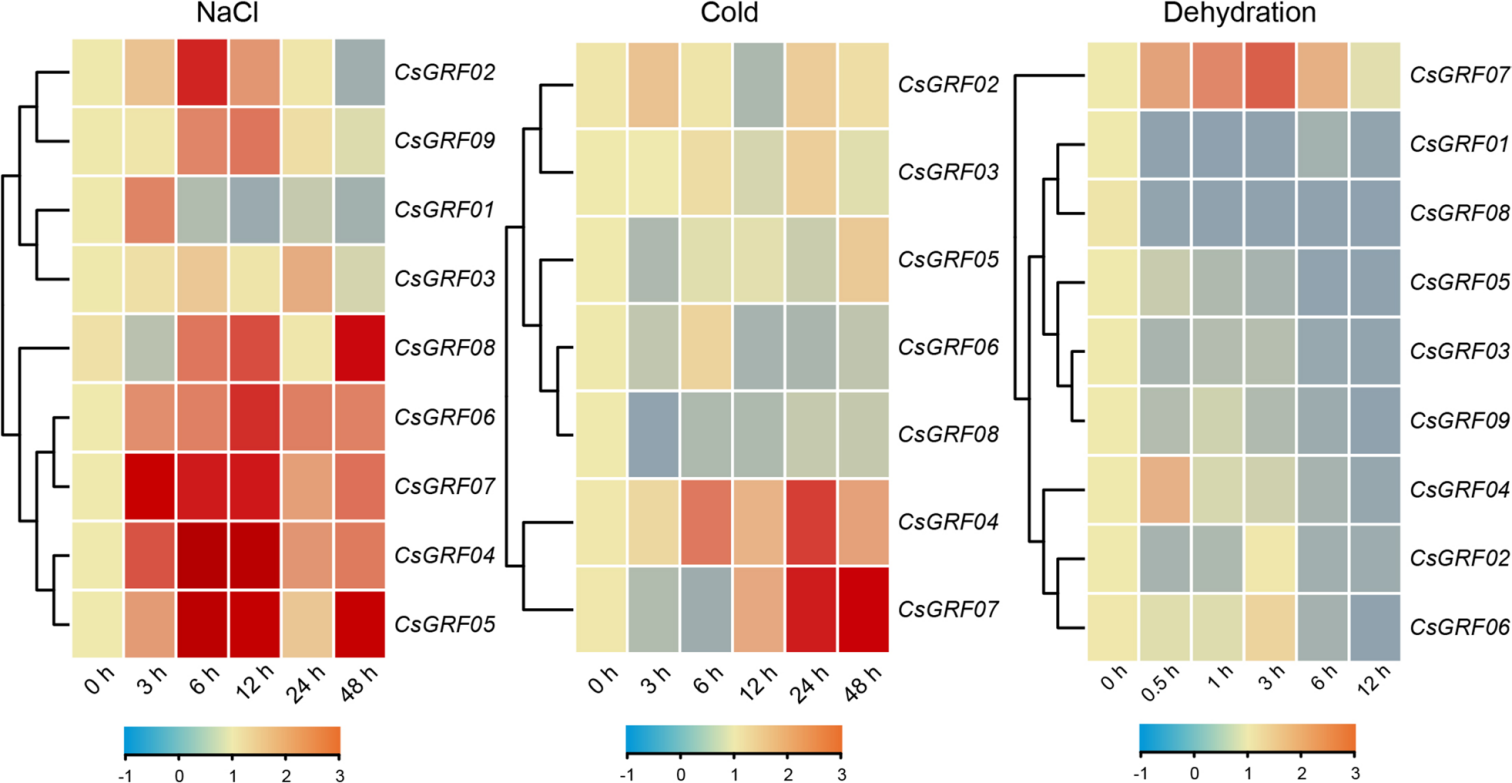
Expression profiles of *CsGRF*s under multiple abiotic stresses. Expression analysis was carried out in leaves of *C*. *sinensis* at different time points (0 h, 3 h, 6 h, 12 h, 24 h and 48 h after NaCl and cold treatments, 0 h, 0.5 h, 1 h, 3 h, 6 h and 12 h after dehydration treatment). The qPCR results of *CsGRFs* were normalized by log_2_ transform. The heatmap constructed by TBtools software. Color scale erected horizontally at the bottom of the diagram

Furthermore, efforts were made to analyze expression patterns of 9 *CsGRFs* under multiple abiotic stresses, namely, salt stress (NaCl), low temperature (Cold) and dehydration. All *CsGRFs* except *CsGRF01* showed significantly up-regulation of gene expression under salt stress. Low-temperature treatment remarkably induced a up-regulated expression of *CsGRF04* and *CsGRF07*, as well as a down-regulated expression of *CsGRF06* and *CsGRF08*, with no significant differences in the expression patterns of the remaining genes (*CsGRF01* and *CsGRF09* were undetectable). All *CsGRFs* were notably down-regulated under dehydration treatment, except for *CsGRF04* and *CsGRF07*, which were up-regulated (Fig. 6, Supplementary Table S6).

### Screening of *CsGRFs* in response to multiple stresses

**Fig. 7:**
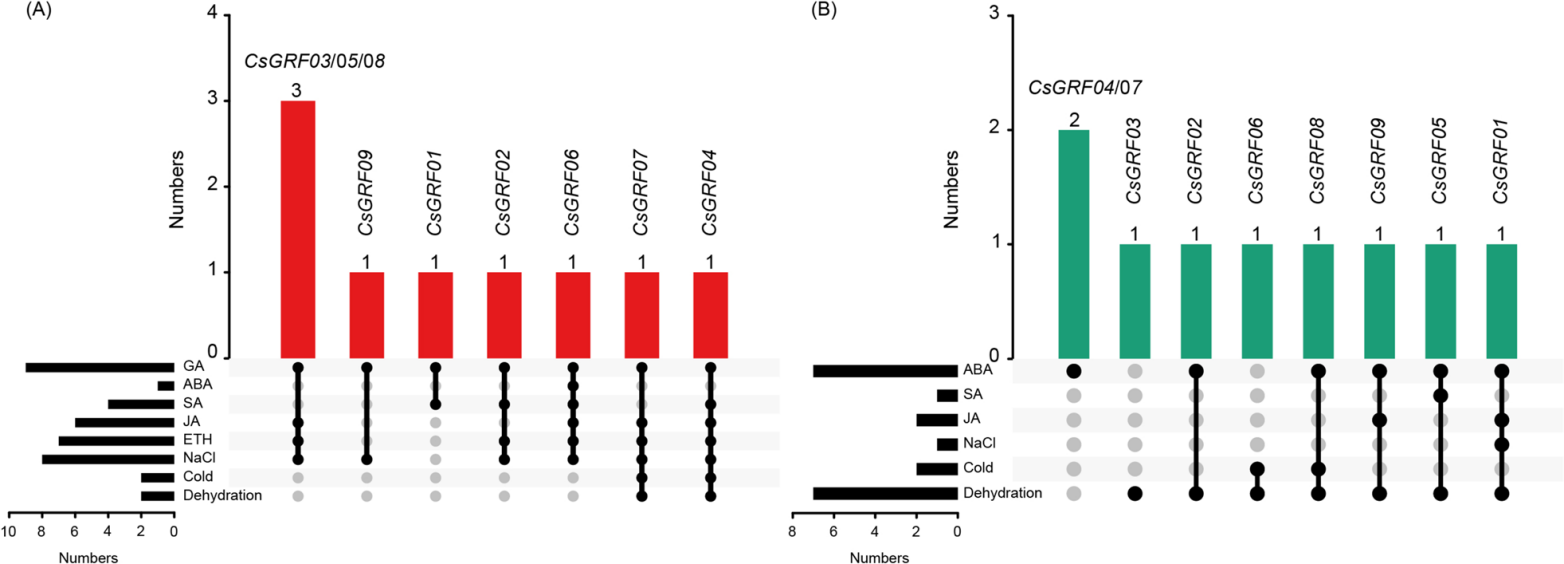
Venn diagram of *CsGRFs* under phytohormone treatments and abiotic stresses. **A** Diagram of overlapping *CsGRFs* which showed up-regulated expression levels under phytohormone treatments and abiotic stresses. The red columns represent the number of overlapping treatments with up-regulated expression pattern under phytohormone treatments and abiotic stresses. The black columns in the lower left corner represent the number of up-regulated *CsGRFs* under each treatments. The black circles strung with lines represent the overlapping treatments. **B** Diagram of overlapping *CsGRFs* which showed down-regulated expression levels under phytohormone treatments and abiotic stresses. The green columns represent the number of overlapping treatments with down-regulated expression pattern under phytohormone treatments and abiotic stresses. The black columns in the lower left corner represent the number of down-regulated *CsGRFs* under each treatments. The black circles strung with lines represent the overlapping treatments

To identify *CsGRFs* capable of responding to multiple stresses, we combined the expression patterns of *CsGRFs* in response to phytohormones with those of abiotic stress treatments, and venn diagrams for *CsGRFs*-up-regulated and *CsGRFs*-down-regulated genes were conducted, separately. As shown in Fig. 7A, among the *CsGRFs*-up-regulated genes expressed after 5 phytohormone and 3 abiotic stress treatments, a *CsGRF04* with significantly up-regulated expression pattern in 7 treatments except ABA treatment was screened out. In addition, *CsGRF06* was up-regulated and induced by 6 treatments except low temperature and dehydration, and *CsGRF07* displayed a markedly up-regulation by 6 treatments except ABA and SA. Conversely, among the *CsGRFs*-down-regulated genes by the 8 treatments, we observed a *CsGRF01* that responded to a total of 4 treatments, which was ABA, JA, NaCl, and dehydration. Simultaneously, *CsGRF05* was found to be down-regulated in response to ABA, SA and dehydration treatments, *CsGRF08* exhibited down-regulated expression patterns after ABA, low temperature and dehydration treatments, along with *CsGRF09* showing down-regulated expression levels under ABA, JA and dehydration treatments (Fig. 7B). In conclusion, we selected *CsGRF04* which responded to the highest number of stresses among all treatment groups, for subsequent functional characterization studies.

### Obtainment of *CsGRF04*-VIGS plants

**Fig. 8:**
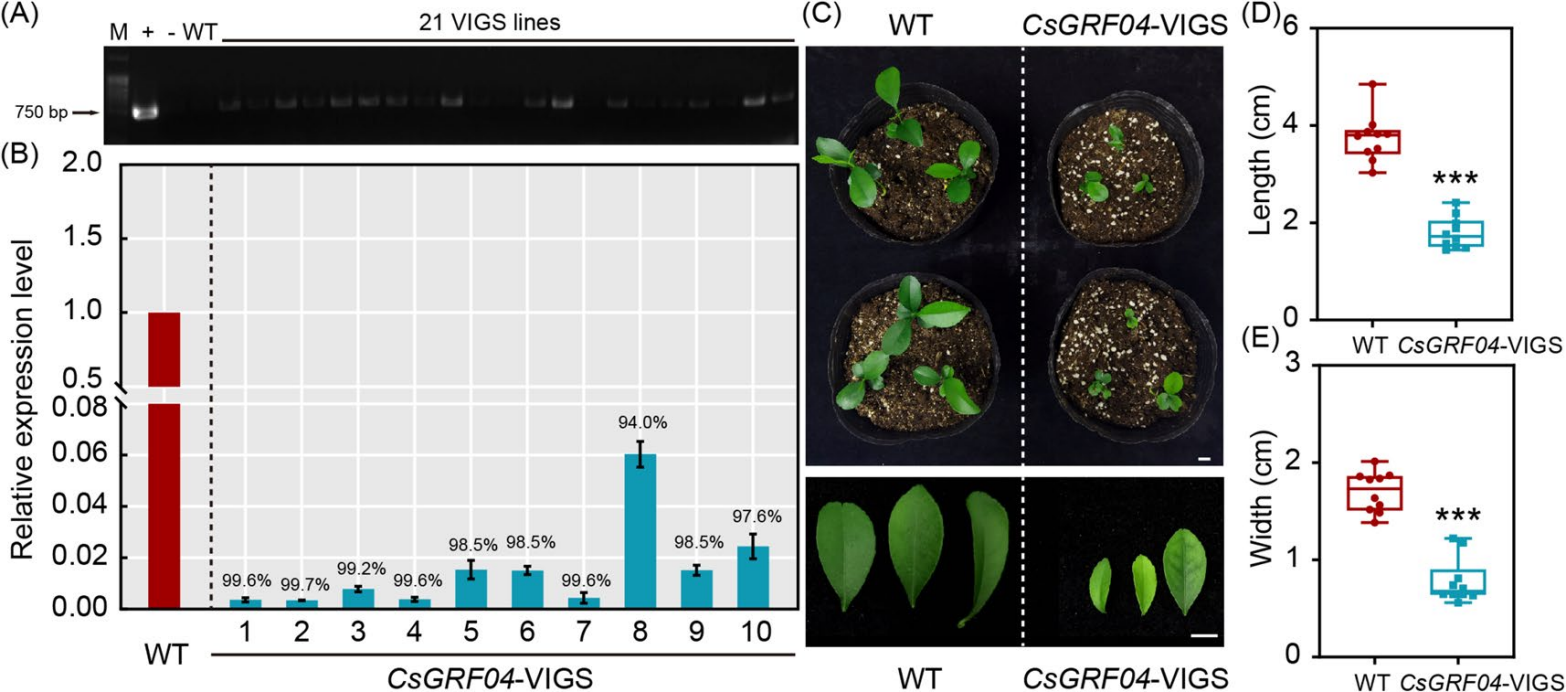
Identification, expression analysis and phenotypic observation of *CsGRF04*-VIGS transgenic plants. **A** Genomic PCR for identification of the *CsGRF04*-VIGS plants. Full-length gels are presented in Supplementary Figure S2. **B** Expression of *CsGRF04* in ten randamly selected positive VIGS lines, as analyzed by qPCR. **C** Phenotypic observation of WT and *CsGRF04-VIGS* plants. **D**, **E** Length (**D**) and width (**E**) statistics of leave from WT and *CsGRF04*-VIGS plants. The asterisk indicates the significant difference between WT and the *CsGRF04*-VIGS plants based on a Tukey’s test (*** *p* < 0.001). The scale bar indicates 1 cm

Virus induced gene silencing (VIGS) is a reversed genetics technology that has been widely used in recent years. By introducing recombinant viral vectors with target genes into host plants, it inhibits the expression of endogenous genes in plants, resulting in the loss of function or reduction of the expression level of the target genes. The VIGS system was employed to silence *CsGRF04* in *C. sinensis* by *Agrobacteri*um-mediated infestation. After genomic PCR identification, 21 strains of *CsGRF04*-VIGS positive plants were identified (Fig. 8A, Full-length gels are presented in Supplementary Figure S2). Ten positive silencing plants randomly selected from *CsGRF04*-VIGS were used for qRT-PCR analysis. The results showed that the expression levels of *CsGRF04* were suppressed by 94.0% to 99.7%, compared with the control plants (Fig. 8B), demonstrating that the silencing of *CsGRF04* in *C. sinensis* using VIGS was successful and effective. Interestingly, we found that after silencing of *CsGRF04*, *C. sinensis* exhibited a significant plant dwarf phenotype compared to the control (Fig. 8C), and the length and width of the leaves were both markedly smaller than those of the control (Fig. 8D, E).

### Identification of abiotic stress resistance in *CsGRF04*-VIGS plants

**Fig. 9:**
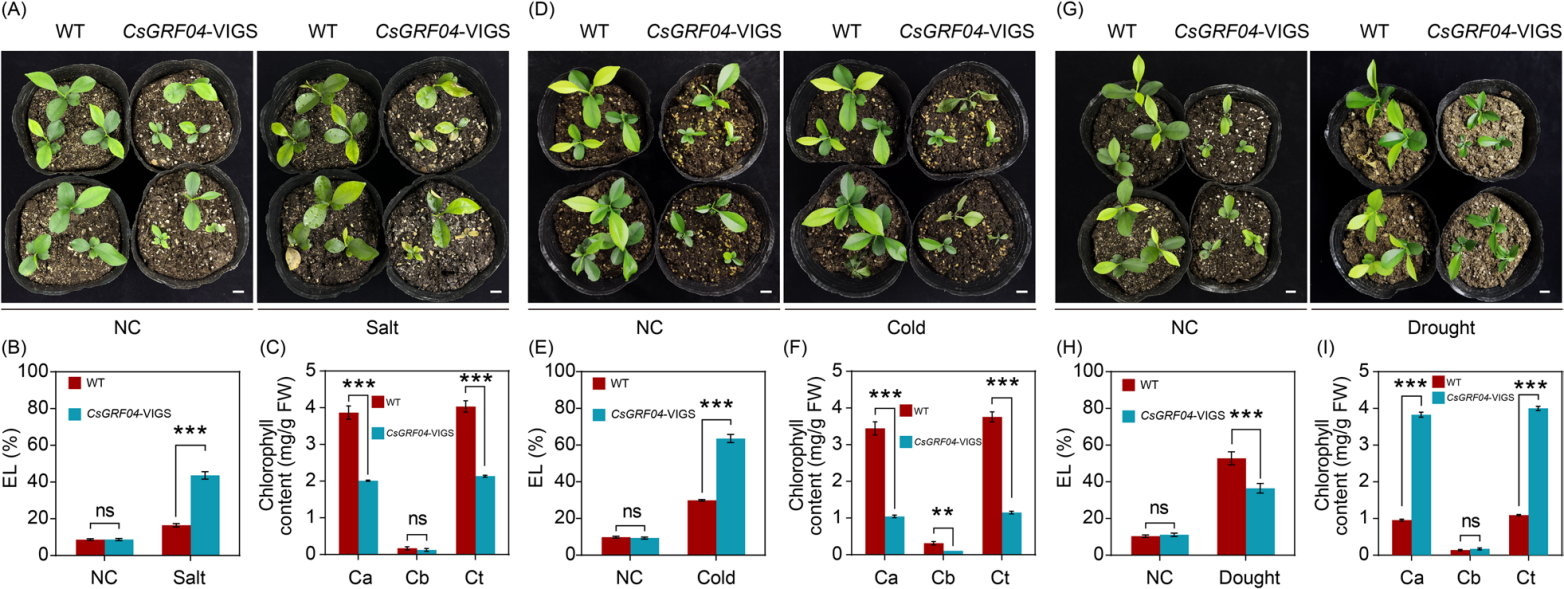
Silencing of *CsGRF04* by virus-induced gene silencing (VIGS) alters abiotic stress resistance in *C. sinensis*. **A** Phenotype of 1-month-old WT and *CsGRF04*-VIGS plants before (left panels) and after (right panels) salt treatment. NC: normal condition. **B**, **C** Electrolyte leakage (EL) (**B**) and chlorophyll content (**C**) of WT and *CsGRF04*-VIGS plants before and after the salt treatment. **D** Phenotype of 1-month-old WT and *CsGRF04*-VIGS plants under NC (left panels) and after recovery (right panels) of cold treatment (8 h at -4 °C and 3 days at ambient temperature). **E**, **F** EL (**E**) and chlorophyll content (**F**) of WT and *CsGRF04*-VIGS plants before and after the cold treatment. **G** Phenotype of 1-month-old WT and *CsGRF04*-VIGS plants before (left panels) and after (right panels) drought treatment. **H**, **I** EL (**H**) and chlorophyll content (**I**) of WT and *CsGRF04*-VIGS plants before and after the drought treatment. Error bars represent ± SE (*n* = 3). ns: not significant. Asterisks indicate significant differences between *CsGRF04*-VIGS and WT plants (***P < 0.001). The scale bar indicates 1 cm

To characterize whether silencing *CsGRF04* could alter the resistance to abiotic stresses in *C. sinensis*, we subjected the *CsGRF04*-VIGS and the WT plants to 300 mM NaCl (Salt), -4 ℃ (Cold), and drought treatments, respectively. Under normal conditions (NC), the WT and *CsGRF04*-VIGS plants were morphologically indistinguishable except for plant size. After 2 weeks of salt treatment, although all treated plants were damaged, *CsGRF04*-VIGS plants showed more severe lesions than the WT, and the symptoms of wilting and waterlogging were all more pronounced, with some of the leaves dying completely, while most of the leaves from WT remained alive (Fig. 9A). Electrolyte leakage (EL) is an important indicator of cell membrane permeability, the larger the value, the more permeation of electrolytes, indicating the more severe damage to the cell membrane. Under NC, there was no apparent difference between the EL of WT and *CsGRF04*-VIGS plants, whereas, the EL of *CsGRF04*-VIGS was significantly higher than that of the WT (Fig. 9B). Before treatment, there was no significant difference in chlorophyll content between WT and *CsGRF04*-VIGS plants, nevertheless, the chlorophyll content of *CsGRF04*-VIGS plants was significantly lower than that of the WT after salt stress (Supplementary Figure S3, Fig. 9C), suggesting that silencing of *CsGRF04* remarkably reduced the salt stress resistance in *C. sinensis*.

Similarly, after 8 h of treatment at -4 °C and 3 days at ambient temperature, all plants were injured to some extent. However, *CsGRF04*-VIGS plants displayed more severe wilting and necrosis in comparison with WT (Fig. 9D, phenotypes before recovery was displayed in Supplemental Figure S4). Consistent with the phenotype, EL in *CsGRF04*-VIGS plants was prominently increased, accompanied by significantly lower chlorophyll content, in comparison with WT when subjected to cold treatment (Fig. 9E, F). These results suggest that silencing of *CsGRF04* promotes cold susceptibility in *C. sinensis*.

After 3 weeks of drought treatment, WT plants exhibited leave yellowing, scorched edges, and even death. In contrast, despite the slight leave yellowing, the overall growth of *CsGRF04*-VIGS was better than that of the WT (Fig. 9G). Meanwhile, *CsGRF04*-VIGS plants exhibited lower EL and higher chlorophyll content relative to the WT (Fig. 9H, I ), suggesting that silencing of *CsGRF04* improves drought tolerance in *C. sinensis*.

## Discussion

GRFs are a family of plant-specific TFs that play essential roles in plant growth and development. In this study, 9 *CsGRF* genes were systematically excavated from *Citrus sinensis*, and gene structures, protein motifs, phylogenetic and syntenye relationships of the CsGRFs were then analysed.

### The evolution and characterization of *CsGRFs* in* C. sinensis*

Based on the phylogenetic analysis, the 9 CsGRF members were grouped into four clusters, which is resembles previous taxonomic researches of GRF families. It has been demonstrated that gain or loss events in exons or introns contribute to structural and functional variability in genes [44]. Regarding the relative gene structures within each cluster, most of the *CsGRF* genes showed a similar gene structure with 2–4 exons, which is consistent with that of Arabidopsis and rice [7, 8]. The QLQ and WRC domains are highly conserved among all CsGRF proteins, and the features of conserved motifs were conservative among same clusters, suggesting that the evolution of the structure and motifs of *CsGRF* genes is conserved to some extent.

Gene duplication events, which including tandem duplication events, segmental duplication events and whole-genome duplication (WGD) events, serve as the major drivers of genome and genetic system evolution [45]. Most of the angiosperms have undergone at least one WGD event in their evolutionary history [46]. Expansion of gene families reflects the effects of WGD, together with tandem and segmental duplications. In this study, only one pair of *CsGRFs* (*CsGRF04* and *CsGRF06*) located on chromosome 5 among the 9 *CsGRF* genes exhibited segmental duplication events (Fig. 3A), and no tandem duplication events were identified, indicating that segmental duplication events may dominate the early expansion of the *CsGRF* family.

After identifying non-redundant *GRF* genes with colinearity between *C. sinensis* and the two model species (Arabidopsis and rice), 9 pairs of colinear *GRF* genes were observed between *C. sinensis* and *A*. *thaliana*, while only 2 pairs of colinear *GRF* genes were detected between *C. sinensis* and *O*. *sativa* (Fig. 3B), suggesting that *C. sinensis* and *A*. *thaliana* shared a strong linear homologous relationship than between *C. sinensis* and *O*. *sativa*. This may be related to the fact that *C. sinensis* and *A*. *thaliana* belong to the same group of dicotyledonous plants and are more closely related evolutionarily. Of note, no evident correlation was found between the number of *GRF* genes and genome dimensions. Although the size of the *C. sinensis* genome (322 Mb) is 2.8 times larger than that of the *A*. *thaliana* genome (116 Mb) [4, 47], they contain the same number of *GRF* members. This suggests that the *C. sinensis* genome may have lost genes during replication.

### *CsGRFs* are involved in phytohormone responses of* C. sinensis*

Previous studies have shown that phytohormones regulate a variety of physiological processes in growth, differentiation, development and environmental adaptation. The first GRF to be identified was OsGRF1 in gibberellin-treated rice [6]. Subsequently, increasing studies have demonstrated that *GRF* genes play multiple and diverse roles in plant responses to phytohormones [48, 49]. In present study, we examined the response of *CsGRF* genes to various phytohormones. All *CsGRFs* showed markedly up-regulated expression levels after GA treatment, with the highest expression reaching 151.4-fold (*CsGRF04*), which result is consistent with the conclusion that GRFs are positive regulators of gibberellin production as found in previous studies [50]. ABA is thought to play a pivotal role in the integration of multiple stress signals (e.g., salinity, drought, and cold) and the control of downstream stress responses in plants. The expression of most *CsGRF* genes showed remarkable down-regulation levels after ABA treatment except for *CsGRF06*, whereas there was no significant difference in the expression of *CsGRF03* (Fig. 5). These results imply that *CsGRF* genes may be involved in abiotic stress tolerance through both ABA-dependent and ABA-independent signaling pathways. The *cis*-element analysis showed that the promoters of *CsGRFs* with ABA-induced expression mostly contained ABRE elements (*CsGRF02*/*04*/*07*/*09*) (Fig. 4), and we therefore hypothesized that *cis*-element analysis could predict the response of certain TFs to hormone treatment. SA and JA are essential endogenous signals in the plant systemic acquired resistance (SAR) signaling pathway. Numerous studies have shown that both SA and JA induce the synthesis of protease inhibitors, nutrient storage proteins, pathogen-associated proteins (PRs), and the expression of protein synthesis genes, thereby modulating plant disease resistance responses [51, 52]. The expression of four *CsGRFs* was significantly up-regulated after SA treatment (C*sGRF01/02/04/06*), *CsGRF05* showed a down-regulation-induced pattern, and the remaining *CsGRFs* displayed non-significant differences in expression. Most of the C*sGRFs* were remarkably up-regulated after JA treatment, except for *CsGRF01* and *CsGRF09*, which showed down-regulated patterns. Notably, we found that *CsGRF01* was markedly up-regulated (30.6-fold) after SA treatment but extremely down-regulated (0.35-fold) after JA treatment; while *CsGRF05* showed the opposite trend. It showed a notable down-regulated expression level (0.28-fold) after SA treatment but a clear up-regulated expression level (3.3-fold) after JA treatment, suggesting that *CsGRF01* and *CsGRF05* may act as mutual antagonists in the immune signaling pathway response to the two plant-defense-related phytohormones, SA and JA (Fig. 5). Ethylene plays an important regulatory role in fruit development and ripening. After ETH treatment, all *CsGRFs* displayed different degrees of up-regulated expression at the transcriptional level, except for *CsGRF01* and *CsGRF09*, whose expression was undetectable (Fig. 5), suggesting that most of the *CsGRFs* could respond positively to ethylene.

### *CsGRFs* are involved in abiotic stresses responses of *C. sinensis*

During the long-term evolutionary process, plants have acquired a series of signaling pathways and defense systems against environmental stresses, and TFs play a crucial role in the response of plants to various adversity stresses [53, 54]. It has been demonstrated that GRF TFs play a critical role in plant adversity stress by coordinating stress response and defense signals [55]. Expression pattern analysis showed that the expression of eight *CsGRFs* was significantly up-regulated and one *CsGRF* was down-regulated under salt treatment. Two *CsGRFs* were extremely up-regulated and two *CsGRFs* were down-regulated under cold treatment. Seven *CsGRFs* were markedly up-regulated and two *CsGRFs* were down-regulated under dehydration treatment (Fig. 6). Evidently, all *CsGRFs* responded to both salt and dehydration treatments, suggesting that they may play an essential role in response to osmotic stress in *C. sinensis*. However, the trends of expression changes of *CsGRFs* under these two treatments were different, indicating that the functions played by different *CsGRFs* in response to osmotic stress may have varied as well. In addition, the transcript abundance of most *CsGRFs* peaked at 3 h or 6 h after abiotic stress treatments, suggesting that *CsGRFs* respond more rapidly to abiotic adversity. Collectively, *CsGRFs* may be involved in biological processes related to abiotic stress response, especially in the response of *C. sinensis* to osmotic stress. While the response and function of *CsGRFs* under abiotic stress need to be further verified.

### Silencing of *CsGRF04* significantly reduced resistance of salt stress and cold stress, but increased drought tolerance in in *C. sinensis*

Combining the expression patterns after multiple phytohormone treatments and abiotic stress treatments, we screened out *CsGRF04*, which responded to the highest number of treatments (significantly up-regulated after 7 treatments), and obtained *CsGRF04*-VIGS lines with markedly reduced *CsGRF04* expression to characterize its function under abiotic stress. It was observed that the *CsGRF04*-VIGS plants exhibited dramatic dwarfing compared to the WT, and the leaf length and width were both obviously lower than that of the WT (Fig. 8). Previous studies have shown that *GRFs* usually play positive roles in plant growth and development. Overexpression of *GRFs* resulted in cell proliferation and leaf expansion in Arabidopsis, maize, tomato and lettuce [13, 56–58]. However, *GRFs* have also been reported to play negative regulatory roles in plant growth and development as well. Overexpression of maize *ZmGRF10* and Arabidopsis *AtGRF9* leads to reduced cell proliferation and plant formation of smaller leaves [59, 60], suggesting the functional diversity of *GRFs* in plant growth. In the present study, plants silenced with *CsGRF04* exhibited smaller leaves and shorter heights, indicating that *CsGRF04* plays a positive role in regulating leaf development in *C. sinensis*. Interestingly, we found that *AtGRF9* is the homologous gene of *CsGRF04* in *A*. *thaliana*. However, their regulatory patterns for leaf growth exhibited opposite trends. This may be due to the existence of differential regulatory networks among different plant species.

Three different abiotic stress treatments to *CsGRF04*-VIGS plants revealed that silencing of *CsGRF04* resulted in reduced resistance to salt stress and cold stress, and increased tolerance to drought stress in *C. sinensis*. Researches have shown that leave size plays an important role in their drought tolerance. The smaller the leave blade, the smaller the area of transpiration water loss, and the stronger the drought tolerance it is [61]. Therefore, we hypothesized that the increased tolerance of *CsGRF04*-VIGS plants to drought stress might be related to their reduced leave size. However, more in-depth studies are needed to elucidate the deeper function and mechanism of *CsGRF04* in different abiotic stresses. Our results contribute comprehensive information for functional studies of *CsGRFs*, provide references for screening phytohormone-responsive and abiotic stress-resistant *CsGRFs*, and lay the foundation for unraveling the molecular mechanisms and regulatory networks in *CsGRFs*.

## Conclusions

A total of 9 *CsGRF* genes were identified and analyzed in *C. sinensis*, including their physical location, phylogenetic relationships, conserved domains, synteny relationships and promoter elements. The qRT-PCR analysis revealed that different *CsGRFs* exhibited multiple response patterns after 5 phytohormone treatments (ABA, GA, SA, JA and ETH) and 3 abiotic stress treatments (NaCl, cold and dehydration). *CsGRF04*, which responded to the highest number of above treatments, was silenced by VIGS and analyzed for resistance to multiple abiotic stresses. The results demonstrated that silencing of *CsGRF04* significantly reduced resistance of salt stress and cold stress, but increased drought tolerance in in *C.* *sinensis*.

### Supplementary Information

**Additional file 1: Table S1.** List of primer sequences used in this study.

**Additional file 2: Table S2.** Proposed nomenclature and important features of CsGRFs.

**Additional file 3: Table S3.** Conserved motifs in CsGRFs proteins.

**Additional file 4: Fig. S1.** Multiple sequence alignment (A) and composition (B-C) of conserved domains in CsGRFs.

**Additional file 5: Table S4.** List of stress-responsive *cis*-acting elements present in 2 kb upstream region of *CsGRFs*.

**Additional file 6: Table S5.** qRT-PCR values of *CsGRFs* under multiple phytohormone treatments

**Additional file 7: Table S6.** qRT-PCR values of *CsGRFs* under multiple abiotic stresses

**Additional file 8: Fig. S2.** The uncropped gel of Fig. 8A. The white blocks indicate where they were cropped.

**Additional file 9: Fig. S3.** The chlorophyll content of WT and *CsGRF04*-VIGS plants before treatments. ns: not significant.

**Additional file 10: Fig. S4.** Phenotype of 1-month-old WT and *CsGRF04*-VIGS plants under normal condition (NC) (left panels), after cold treatment (8 h at -4 °C) (middle panels) and after 3 days of recovery (right panels). The scale bar indicates 1 cm.

## Data Availability

The sequence information of *Citrus sinensis* GRF family genes were collected from Citrus Pan-genome to Breeding Database (http://citrus.hzau.edu.cn/index.php), and the GRF protein sequences of Arabidopsis (*Arabidopsis thaliana*), rice (*Oryza sativa subsp. japonica*), poplar (*Populus trichocarpa*), pear (*Pyrus bretschneideri*) and grape (*Vitis vinifera*) were downloaded from the ensembl website (http://asia.ensembl.org/index.html). All data used during the current study are included in this published article and its supplementary information files or available from the corresponding author on reasonable request.

## Abbreviations

aa: Sequence, amino acid
ABA: Abscisic acid
ETH: Ethrel
GA: Gibberellin
JA: Jasmonic acid
qPCR: Quantitative polymerase chain Reaction
qRT-PCR: Quantitative real-time polymerase chain reaction
SA: Salicylic acid
SAR: Systemic acquired resistance
TF: Transcription factor
