# Supplementary material for: Genome-wide identification of the GRF family in sweet orange (Citrus sinensis) and functional analysis of the CsGRF04 in response to multiple abiotic stresses
Source: BMC Genomics. 2024 Jan 6;25:37. doi: 10.1186/s12864-023-09952-8 (PMC10770916; doi:10.1186/s12864-023-09952-8)
Supplement: Supplementary file 1 — Additional file 1: Table S1. List of primer sequences used in this study. [file 12864_2023_9952_MOESM1_ESM.docx]

**Additional file 1: Table S1. List of primer sequences used in this study**

| **Purpose** | **Primer names** | **Primer sequences (5’-3’)** | |
| --- | --- | --- | --- |
|  |  | **Forward primer** | **Reverse primer** |
| Gene expression | CsActin QRT | CATCCCTCAGCACCTTCC | CCAACCTTAGCACTTCTCC |
|  | CsGRF01 QRT | TGGATCCTGTTCTCAGACGG | TGATGATTTGAAGTCAGTGCCC |
|  | CsGRF02 QRT | TTCCATGATGTCTTCCACCA | CAAGTCCAAACACCGATTGA |
|  | CsGRF03 QRT | ATAGGAGCAGCATTGGCAGT | AGCAGCCATCTGTGACTGTG |
|  | CsGRF04 QRT | TCCCAGTACAAGCTCTGCAA | AAATTCGGCGAGGGATTACT |
|  | CsGRF05 QRT | CAGCGGCTACATGACCAATA | TCCTCATCGGCCATATTTTC |
|  | CsGRF06 QRT | TTCTACAGAAGGCGCTGGTT | TTGACCAAACCACAGCAAAG |
|  | CsGRF07 QRT | ACCCATGTCATCTGGGTCAT | CTGGGAAATCTGGAGGTTGA |
|  | CsGRF08 QRT | AGTCGAAATCCAGCAACACC | GGCCACTCATCAAAGAAAGG |
|  | CsGRF09 QRT | GATGAATGGCCACCAAAATC | TGAAGAGGGAATGGAAATCG |
| Virus-induced gene silencing | CsGRF04-pTRV2 | GGATCCTGAAGCCCCAAAACCCACCAAC | CCCGGGAATGATCCAGACCCATAAAAGC |
|  | pTRV2 | ATTCACTGGGAGATGATACGCT |  |
