# Supplementary material for: Genome-wide identification of the GRF family in sweet orange (Citrus sinensis) and functional analysis of the CsGRF04 in response to multiple abiotic stresses
Source: BMC Genomics. 2024 Jan 6;25:37. doi: 10.1186/s12864-023-09952-8 (PMC10770916; doi:10.1186/s12864-023-09952-8)
Supplement: Supplementary file 2 — Additional file 2: Table S2. Proposed nomenclature and important features of CsGRFs. [file 12864_2023_9952_MOESM2_ESM.docx]

**Additional file 2: Table S2. Proposed nomenclature and important features of CsGRFs**

| **Gene name** | **Gene ID** | **Chromosome NO.** | **Strand (+ or -)** | **Start position** | **End position** | **Exon number** | **Intron number** | **Open readeing frame** | **Amino acid number** | **Relative molecular weight (kD)** | **Isoelectric point** | **Group** |
| --- | --- | --- | --- | --- | --- | --- | --- | --- | --- | --- | --- | --- |
| CsGRF01 | Cs1g_pb016580.1 | 1 | + | 22256995 | 22259362 | 3 | 2 | 1146 | 381 | 60.23 | 5.48 | I |
| CsGRF02 | Cs1g_pb018790.1 | 1 | + | 24928426 | 24933000 | 4 | 3 | 1803 | 600 | 36.57 | 8.88 | V |
| CsGRF03 | Cs3g_pb009170.2 | 3 | - | 18995187 | 18999256 | 4 | 3 | 1086 | 361 | 65.05 | 7.27 | I |
| CsGRF04 | Cs5g_pb006400.1 | 5 | - | 8617373 | 8619395 | 3 | 2 | 699 | 232 | 36.50 | 8.96 | II |
| CsGRF05 | Cs5g_pb014920.1 | 5 | - | 2354041 | 2357479 | 3 | 2 | 1119 | 372 | 39.76 | 9.08 | IV |
| CsGRF06 | Cs5g_pb019860.2 | 5 | + | 19550806 | 19556362 | 2 | 1 | 1665 | 554 | 40.92 | 7.32 | IV |
| CsGRF07 | Cs6g_pb011420.1 | 6 | - | 17413709 | 17417078 | 4 | 3 | 1224 | 407 | 42.18 | 8.46 | IV |
| CsGRF08 | Cs7g_pb011260.1 | 7 | - | 8774152 | 8778713 | 3 | 2 | 999 | 332 | 25.26 | 9.75 | I |
| CsGRF09 | CsUn_pb049640.3 | Un | - | 56885781 | 56888040 | 3 | 2 | 969 | 322 | 44.68 | 8.49 | I |
