# Supplementary material for: Genome-wide identification of the GRF family in sweet orange (Citrus sinensis) and functional analysis of the CsGRF04 in response to multiple abiotic stresses
Source: BMC Genomics. 2024 Jan 6;25:37. doi: 10.1186/s12864-023-09952-8 (PMC10770916; doi:10.1186/s12864-023-09952-8)
Supplement: Supplementary file 3 — Additional file 3: Table S3. Conserved motifs in CsGRFs proteins. [file 12864_2023_9952_MOESM3_ESM.docx]

**Additional file 3: Table S3. Conserved motifs in CsGRFs proteins**

| **Motif** | **Width** | **E-value** | **Consensus Sequence** |
| --- | --- | --- | --- |
| Motif 1^1^ | 47 | 4.1e-281 | DPEPGRCRRTDGKKWRCSKDAYPDSKYCERHMHRGRNRSRKPVELQT |
| Motif 2^2^ | 37 | 4.1e-141 | PFTASQWQELEHQALIYKYMVAGLPVPPDLLLPIKKS |
| Motif 3^3^ | 9 | 7.2e-015 | LRHFFDEWP |
| Motif 4^4^ | 10 | 1.8e-004 | TTKLSISIPI |
| Motif 5 | 20 | 3.5e-004 | FPPQAPHVEWGCFQVGFGRK |
| Motif 6 | 16 | 2.8e-004 | FHHPTFGYCSFFGKKI |
| ^1^Conserved sequences within the WRC domain; ^2^QLQ motif; ^3^FFD domain; ^4^TQL domain. | | | |

**Additional file 3: Table S3. Conserved motifs in CsGRFs proteins**

| **Motif** | **Width** | **E-value** | **Consensus Sequence** |
| --- | --- | --- | --- |
| Motif 1^1^ | 47 | 4.1e-281 | DPEPGRCRRTDGKKWRCSKDAYPDSKYCERHMHRGRNRSRKPVELQT |
| Motif 2^2^ | 37 | 4.1e-141 | PFTASQWQELEHQALIYKYMVAGLPVPPDLLLPIKKS |
| Motif 3^3^ | 9 | 7.2e-015 | LRHFFDEWP |
| Motif 4^4^ | 10 | 1.8e-004 | TTKLSISIPI |
| Motif 5 | 20 | 3.5e-004 | FPPQAPHVEWGCFQVGFGRK |
| Motif 6 | 16 | 2.8e-004 | FHHPTFGYCSFFGKKI |
| ^1^Conserved sequences within the WRC domain; ^2^QLQ motif; ^3^FFD domain; ^4^TQL domain. | | | |
