# Supplementary material for: Genome-wide identification of the GRF family in sweet orange (Citrus sinensis) and functional analysis of the CsGRF04 in response to multiple abiotic stresses
Source: BMC Genomics. 2024 Jan 6;25:37. doi: 10.1186/s12864-023-09952-8 (PMC10770916; doi:10.1186/s12864-023-09952-8)
Supplement: Supplementary file 4 — Additional file 4: Fig. S1. Multiple sequence alignment (A) and composition (B-C) of conserved domains in CsGRFs. [file 12864_2023_9952_MOESM4_ESM.docx]

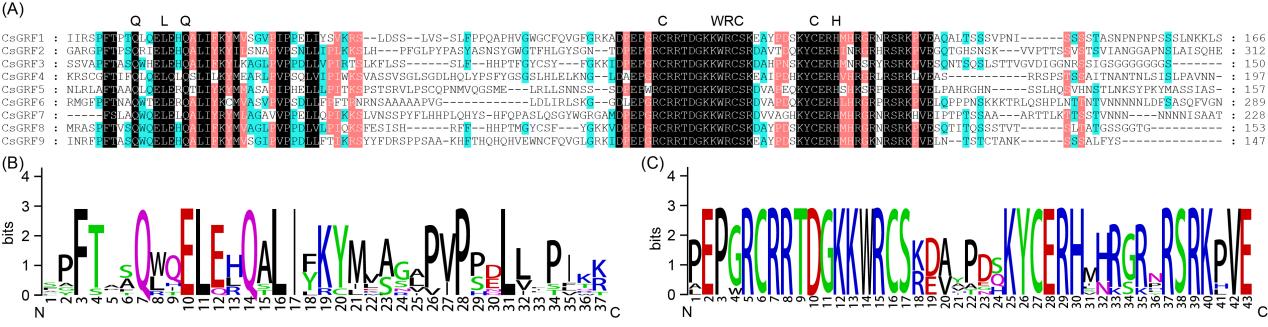


**Additional file 4: Fig. S1. Multiple sequence alignment (A) and composition (B-C) of conserved domains in CsGRFs.**
