# Supplementary material for: Genome-wide identification of the GRF family in sweet orange (Citrus sinensis) and functional analysis of the CsGRF04 in response to multiple abiotic stresses
Source: BMC Genomics. 2024 Jan 6;25:37. doi: 10.1186/s12864-023-09952-8 (PMC10770916; doi:10.1186/s12864-023-09952-8)
Supplement: Supplementary file 5 — Additional file 5: Table S4. List of stress-responsive cis-acting elements present in 2 kb upstream region of CsGRFs. [file 12864_2023_9952_MOESM5_ESM.docx]

**Additional file 5: Table S4. List of stress-responsive *cis*-acting elements present in 2 kb upstream region of *CsGRFs***

| **Gene name** | ***cis***-element | **Sequence** | **Position** | **Annotation** |
| --- | --- | --- | --- | --- |
| *CsGRF01* | MYC | TCTCTTA | 936 | MYC TF-binding site |
| *CsGRF01* | MYC | TCTCTTA | 2375 | MYC TF-binding site |
| *CsGRF01* | MYC | CATTTG | 1685 | MYC TF-binding site |
| *CsGRF01* | MYC | CATTTG | 2090 | MYC TF-binding site |
| *CsGRF01* | MYB | TAACCA | 63 | MYB TF-binding site |
| *CsGRF01* | MYB | CAACAG | 2186 | MYB TF-binding site |
| *CsGRF01* | MYB | CAACCA | 2274 | MYB TF-binding site |
| *CsGRF01* | W-box | TTGACC | 1899 | WRKY TF-binding site |
| *CsGRF02* | MYB | CCGTTG | 1890 | MYB TF-binding site |
| *CsGRF02* | MYB | CAACAG | 382 | MYB TF-binding site |
| *CsGRF02* | MYB | TAACCA | 1780 | MYB TF-binding site |
| *CsGRF02* | MYB | TAACTG | 1919 | MYB TF-binding site |
| *CsGRF02* | ABRE | ACGTG | 174 | *cis*-acting element involved in the abscisic acid responsiveness |
| *CsGRF02* | ABRE | CACGTG | 194 | *cis*-acting element involved in the abscisic acid responsiveness |
| *CsGRF02* | ABRE | ACGTG | 663 | *cis*-acting element involved in the abscisic acid responsiveness |
| *CsGRF02* | ABRE | ACGTG | 1804 | *cis*-acting element involved in the abscisic acid responsiveness |
| *CsGRF03* | MYC | CAATTG | 933 | MYC TF-binding site |
| *CsGRF03* | MYC | CAATTG | 1202 | MYC TF-binding site |
| *CsGRF03* | MYB | CAACAG | 77 | MYB TF-binding site |
| *CsGRF03* | MYB | CAACCA | 1946 | MYB TF-binding site |
| *CsGRF04* | MYC | CATTTG | 2108 | MYC TF-binding site |
| *CsGRF04* | MYB | CAACCA | 1643 | MYB TF-binding site |
| *CsGRF04* | MYB | TAACCA | 1726 | MYB TF-binding site |
| *CsGRF04* | MYB | CAACAG | 2061 | MYB TF-binding site |
| *CsGRF04* | ABRE | TACGGTC | 41 | *cis*-acting element involved in the abscisic acid responsiveness |
| *CsGRF04* | ABRE | ACGTG | 598 | *cis*-acting element involved in the abscisic acid responsiveness |
| *CsGRF04* | ABRE | ACGTG | 937 | *cis*-acting element involved in the abscisic acid responsiveness |
| *CsGRF04* | MYB | TAACTG | 2242 | MYB TF-binding site |
| *CsGRF04* | W-box | TTGACC | 2468 | WRKY TF-binding site |
| *CsGRF05* | MYC | CAATTG | 1116 | MYC TF-binding site |
| *CsGRF05* | MYB | TAACCA | 1596 | MYB TF-binding site |
| *CsGRF05* | MYB | CAACAG | 2111 | MYB TF-binding site |
| *CsGRF05* | MYB | TAACTG | 1993 | MYB TF-binding site |
| *CsGRF06* | MYB | CAACAG | 1092 | MYB TF-binding site |
| *CsGRF06* | MYB | CCGTTG | 919 | MYB TF-binding site |
| *CsGRF06* | MYB | CAACTG | 2270 | MYB TF-binding site |
| *CsGRF07* | ABRE | ACGTG | 201 | *cis*-acting element involved in the abscisic acid responsiveness |
| *CsGRF07* | MYC | CAATTG | 936 | MYC TF-binding site |
| *CsGRF07* | MYC | CAATTG | 979 | MYC TF-binding site |
| *CsGRF07* | MYB | CAACCA | 2443 | MYB TF-binding site |
| *CsGRF08* | MYB | CAACTG | 470 | MYB TF-binding site |
| *CsGRF08* | MYB | CAACAG | 780 | MYB TF-binding site |
| *CsGRF08* | MYB | TAACCA | 1472 | MYB TF-binding site |
| *CsGRF08* | MYC | TCTCTTA | 589 | MYC TF-binding site |
| *CsGRF08* | MYC | CAATTG | 1206 | MYC TF-binding site |
| *CsGRF08* | MYC | CATTTG | 1872 | MYC TF-binding site |
| *CsGRF09* | MYB | CAACCA | 18 | MYB TF-binding site |
| *CsGRF09* | MYB | TAACCA | 946 | MYB TF-binding site |
| *CsGRF09* | MYC | CATTTG | 1083 | MYC TF-binding site |
| *CsGRF09* | MYC | CATGTG | 1667 | MYC TF-binding site |
| *CsGRF09* | ABRE | ACGTG | 329 | *cis*-acting element involved in the abscisic acid responsiveness |
| *CsGRF09* | MYB | CCGTTG | 2242 | MYB TF-binding site |
| *CsGRF09* | MYB | TAACCA | 946 | MYB TF-binding site |
| *CsGRF01* | CAT-box | GCCACT | 278 | *cis*-acting regulatory element related to meristem expression |
| *CsGRF02* | CAT-box | GCCACT | 231 | *cis*-acting regulatory element related to meristem expression |
| *CsGRF07* | CAT-box | GCCACT | 1893 | *cis*-acting regulatory element related to meristem expression |
| *CsGRF05* | HD-Zip 1 | CAAT(A/T)ATTG | 532 | *cis*-element involved in differentiation of the palisade mesophyll cells |
| *CsGRF08* | HD-Zip 1 | CAAT(A/T)ATTG | 426 | *cis*-element involved in differentiation of the palisade mesophyll cells |
| *CsGRF01* | LRE | GAAAGGCAGAC | 605 | *cis*-acting regulatory element involved in light responsiveness |
| *CsGRF01* | LRE | TCTTAC | 938 | *cis*-acting regulatory element involved in light responsiveness |
| *CsGRF02* | LRE | ATAGATAA | 1382 | *cis*-acting regulatory element involved in light responsiveness |
| *CsGRF03* | LRE | GGTTAA | 375 | *cis*-acting regulatory element involved in light responsiveness |
| *CsGRF04* | LRE | GGTTAA | 632 | *cis*-acting regulatory element involved in light responsiveness |
| *CsGRF04* | LRE | GGTTAA | 971 | *cis*-acting regulatory element involved in light responsiveness |
| *CsGRF04* | LRE | GGTTAA | 1551 | *cis*-acting regulatory element involved in light responsiveness |
| *CsGRF06* | LRE | TCTTAC | 1270 | *cis*-acting regulatory element involved in light responsiveness |
| *CsGRF06* | LRE | GATAGGA | 1171 | *cis*-acting regulatory element involved in light responsiveness |
| *CsGRF06* | LRE | GGTTAA | 1987 | *cis*-acting regulatory element involved in light responsiveness |
| *CsGRF08* | LRE | TCTTAC | 1312 | *cis*-acting regulatory element involved in light responsiveness |
| *CsGRF09* | LRE | TCTTAC | 1789 | *cis*-acting regulatory element involved in light responsiveness |
