# Supplementary material for: Genome-wide identification of the GRF family in sweet orange (Citrus sinensis) and functional analysis of the CsGRF04 in response to multiple abiotic stresses
Source: BMC Genomics. 2024 Jan 6;25:37. doi: 10.1186/s12864-023-09952-8 (PMC10770916; doi:10.1186/s12864-023-09952-8)
Supplement: Supplementary file 6 — Additional file 6: Table S5. qRT-PCR values of CsGRFs under multiple phytohormone treatments [file 12864_2023_9952_MOESM6_ESM.docx]

**Additional file 6: Table S5. qRT-PCR values of *CsGRFs* under multiple phytohormone treatments**

| **Treatment** | **Gene name** | **Time points** | | | | | |
| --- | --- | --- | --- | --- | --- | --- | --- |
|  |  | 0 h | 3 h | 6 h | 12 h | 24 h | 48 h |
| **GA** | *CsGRF01* | 1.09 | 1.34 | 0.30 | 16.74 | 4.82 | 0.40 |
|  | *CsGRF02* | 1.01 | 60.98 | 8.91 | 5.13 | 2.69 | 0.49 |
|  | *CsGRF03* | 1.01 | 118.95 | 1.40 | 0.39 | 1.70 | 0.53 |
|  | *CsGRF04* | 1.00 | 151.40 | 1.79 | 0.90 | 0.87 | 0.31 |
|  | *CsGRF05* | 1.02 | 42.82 | 0.06 | 0.26 | 0.87 | 1.11 |
|  | *CsGRF06* | 1.00 | 81.10 | 0.53 | 0.54 | 0.49 | 0.41 |
|  | *CsGRF07* | 1.01 | 43.94 | 3.65 | 0.54 | 0.67 | 0.47 |
|  | *CsGRF08* | 1.01 | 76.35 | 0.75 | 0.60 | 1.43 | 0.74 |
|  | *CsGRF09* | 1.02 | 55.65 | 0.44 | 0.23 | 0.26 | 0.29 |
| **ABA** | *CsGRF01* | 1.06 | 0.26 | 0.13 | 0.06 | 0.77 | 1.07 |
|  | *CsGRF02* | 1.00 | 0.19 | 0.48 | 0.36 | 0.04 | 0.03 |
|  | *CsGRF03* | 1.00 | 0.41 | 0.95 | 0.46 | 1.16 | 1.20 |
|  | *CsGRF04* | 1.02 | 0.11 | 0.26 | 1.42 | 0.94 | 1.04 |
|  | *CsGRF05* | 1.14 | 0.32 | 5.00 | 1.16 | 0.03 | 0.03 |
|  | *CsGRF06* | 1.06 | 1.40 | 4.69 | 3.07 | 3.77 | 2.19 |
|  | *CsGRF07* | 1.06 | 0.55 | 0.26 | 0.32 | 0.42 | 0.98 |
|  | *CsGRF08* | 1.00 | 0.08 | 0.78 | 0.36 | 0.77 | 1.05 |
|  | *CsGRF09* | 1.12 | 0.10 | 0.11 | 0.24 | 0.88 | 1.16 |
| **SA** | *CsGRF01* | 1.05 | 0.53 | 7.37 | 21.86 | 30.63 | 0.23 |
|  | *CsGRF02* | 1.03 | 5.57 | 12.84 | 31.67 | 5.19 | 2.76 |
|  | *CsGRF03* | 1.01 | 0.55 | 1.29 | 1.10 | 1.62 | 0.25 |
|  | *CsGRF04* | 1.00 | 1.26 | 2.32 | 10.65 | 3.74 | 3.13 |
|  | *CsGRF05* | 1.08 | 0.94 | 0.68 | 0.28 | 2.51 | 0.47 |
|  | *CsGRF06* | 1.01 | 0.86 | 1.41 | 1.30 | 3.03 | 0.98 |
|  | *CsGRF07* | 1.01 | 1.26 | 2.11 | 0.28 | 1.30 | 0.62 |
|  | *CsGRF08* | 1.02 | 1.26 | 1.32 | 0.80 | 2.06 | 0.82 |
|  | *CsGRF09* | 1.00 | 0.54 | 1.74 | 0.70 | 0.58 | 1.49 |
| **JA** | *CsGRF01* | 1.00 | 1.25 | 0.35 | 0.47 | 0.62 | 0.89 |
|  | *CsGRF03* | 1.01 | 3.26 | 1.21 | 2.07 | 3.91 | 6.30 |
|  | *CsGRF04* | 1.04 | 11.74 | 74.60 | 22.21 | 1.60 | 1.20 |
|  | *CsGRF05* | 1.02 | 2.16 | 0.88 | 3.32 | 2.64 | 1.23 |
|  | *CsGRF06* | 1.01 | 3.51 | 2.74 | 6.30 | 2.97 | 1.61 |
|  | *CsGRF07* | 1.01 | 1.80 | 5.68 | 3.35 | 0.87 | 1.28 |
|  | *CsGRF08* | 1.00 | 6.25 | 4.02 | 10.21 | 6.59 | 3.11 |
|  | *CsGRF09* | 1.05 | 0.25 | 0.13 | 0.48 | 0.40 | 0.68 |
| **ETH** | *CsGRF02* | 1.04 | 1.71 | 2.19 | 3.59 | 0.06 | 2.19 |
|  | *CsGRF03* | 1.04 | 0.66 | 13.73 | 2.16 | 0.54 | 5.09 |
|  | *CsGRF04* | 1.08 | 1.03 | 11.17 | 4.62 | 0.16 | 4.09 |
|  | *CsGRF05* | 1.02 | 0.20 | 4.90 | 0.54 | 0.42 | 3.40 |
|  | *CsGRF06* | 1.01 | 0.69 | 12.79 | 1.10 | 0.47 | 2.85 |
|  | *CsGRF07* | 1.01 | 3.30 | 9.67 | 3.01 | 1.14 | 18.08 |
|  | *CsGRF08* | 1.05 | 0.60 | 19.54 | 1.88 | 1.01 | 10.37 |
