# Supplementary material for: Genome-wide identification of the GRF family in sweet orange (Citrus sinensis) and functional analysis of the CsGRF04 in response to multiple abiotic stresses
Source: BMC Genomics. 2024 Jan 6;25:37. doi: 10.1186/s12864-023-09952-8 (PMC10770916; doi:10.1186/s12864-023-09952-8)
Supplement: Supplementary file 7 — Additional file 7: Table S6. qRT-PCR values of CsGRFs under multiple abiotic stresses [file 12864_2023_9952_MOESM7_ESM.docx]

**Additional file 7: Table S6. qRT-PCR values of *CsGRFs* under multiple abiotic stresses**

| **Treatment** | **Gene name** | **Time points** | | | | | |
| --- | --- | --- | --- | --- | --- | --- | --- |
|  |  | 0 h | 3 h (0.5 h for dehydration) | 6 h (1 h for dehydration) | 12 h (3 h for dehydration) | 24 h (6 h for dehydration) | 48 h (12 h for dehydration) |
| **NaCl** | *CsGRF01* | 1.02 | 3.22 | 0.28 | 0.09 | 0.48 | 0.15 |
|  | *CsGRF02* | 1.02 | 1.70 | 7.63 | 2.73 | 1.08 | 0.14 |
|  | *CsGRF03* | 1.02 | 1.17 | 1.58 | 1.10 | 2.18 | 0.64 |
|  | *CsGRF04* | 1.01 | 5.10 | 32.50 | 22.26 | 2.73 | 3.59 |
|  | *CsGRF05* | 1.00 | 2.59 | 21.84 | 13.93 | 1.60 | 13.54 |
|  | *CsGRF06* | 1.00 | 2.94 | 3.39 | 7.08 | 3.44 | 3.33 |
|  | *CsGRF07* | 1.00 | 11.34 | 8.31 | 8.53 | 2.47 | 3.92 |
|  | *CsGRF08* | 1.15 | 0.36 | 3.71 | 5.32 | 1.05 | 9.69 |
|  | *CsGRF09* | 1.02 | 1.09 | 3.21 | 3.76 | 1.17 | 0.75 |
| **Cold** | *CsGRF02* | 1.08 | 1.71 | 1.10 | 0.24 | 1.48 | 1.17 |
|  | *CsGRF03* | 1.01 | 0.99 | 1.21 | 0.65 | 1.45 | 0.80 |
|  | *CsGRF04* | 1.03 | 1.29 | 3.59 | 1.99 | 6.12 | 2.42 |
|  | *CsGRF05* | 1.01 | 0.25 | 0.79 | 0.83 | 0.52 | 1.56 |
|  | *CsGRF06* | 1.03 | 0.44 | 1.38 | 0.19 | 0.22 | 0.41 |
|  | *CsGRF07* | 1.04 | 0.28 | 0.12 | 2.24 | 8.27 | 10.67 |
|  | *CsGRF08* | 1.01 | 0.02 | 0.26 | 0.26 | 0.48 | 0.48 |
| **Dehydration** | *CsGRF01* | 1.01 | 0.01 | 0.01 | 0.02 | 0.17 | 0.03 |
|  | *CsGRF02* | 1.01 | 0.20 | 0.25 | 1.04 | 0.15 | 0.12 |
|  | *CsGRF03* | 1.00 | 0.21 | 0.30 | 0.32 | 0.07 | 0.03 |
|  | *CsGRF04* | 1.01 | 2.04 | 0.68 | 0.59 | 0.20 | 0.06 |
|  | *CsGRF05* | 1.00 | 0.51 | 0.27 | 0.20 | 0.03 | 0.01 |
|  | *CsGRF06* | 1.04 | 0.78 | 0.76 | 1.39 | 0.17 | 0.02 |
|  | *CsGRF07* | 1.01 | 2.41 | 3.16 | 4.60 | 2.06 | 0.82 |
|  | *CsGRF08* | 1.10 | 0.03 | 0.01 | 0.01 | 0.02 | 0.00 |
|  | *CsGRF09* | 1.01 | 0.30 | 0.59 | 0.28 | 0.11 | 0.02 |
