# Supplementary material for: Genome-wide identification of the GRF family in sweet orange (Citrus sinensis) and functional analysis of the CsGRF04 in response to multiple abiotic stresses
Source: BMC Genomics. 2024 Jan 6;25:37. doi: 10.1186/s12864-023-09952-8 (PMC10770916; doi:10.1186/s12864-023-09952-8)
Supplement: Supplementary file 8 — Additional file 8: Fig. S2. The uncropped gel of Fig. 8A. The white blocks indicate where they were cropped. [file 12864_2023_9952_MOESM8_ESM.docx]

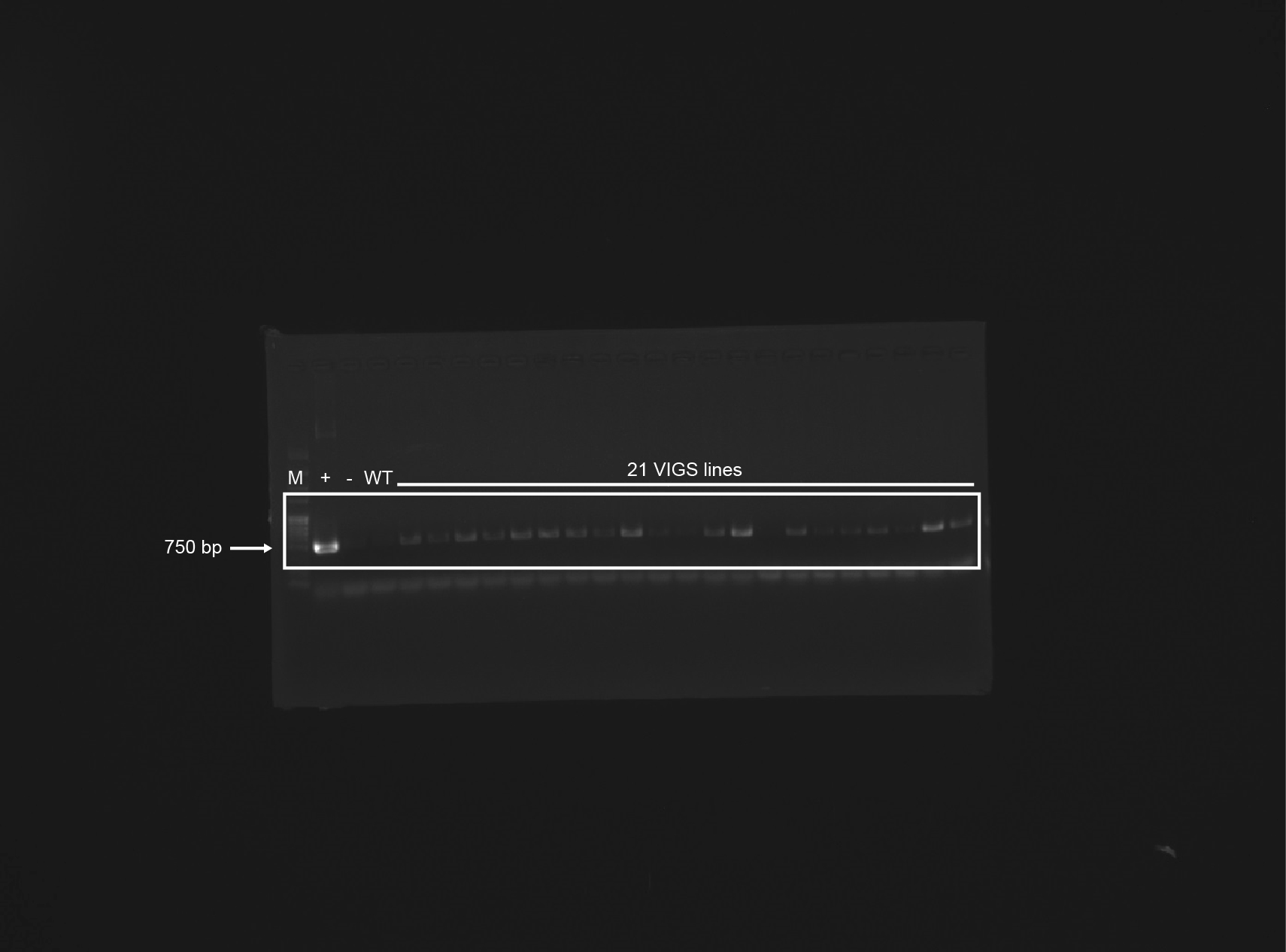


**Additional file 8: Fig. S2. The uncropped gel of Figure 8A.**

The white blocks indicate where they were cropped.
