# Supplementary material for: Genome-wide identification of the GRF family in sweet orange (Citrus sinensis) and functional analysis of the CsGRF04 in response to multiple abiotic stresses
Source: BMC Genomics. 2024 Jan 6;25:37. doi: 10.1186/s12864-023-09952-8 (PMC10770916; doi:10.1186/s12864-023-09952-8)
Supplement: Supplementary file 9 — Additional file 9: Fig. S3. The chlorophyll content of WT and CsGRF04-VIGS plants before treatments. ns: not significant. [file 12864_2023_9952_MOESM9_ESM.docx]

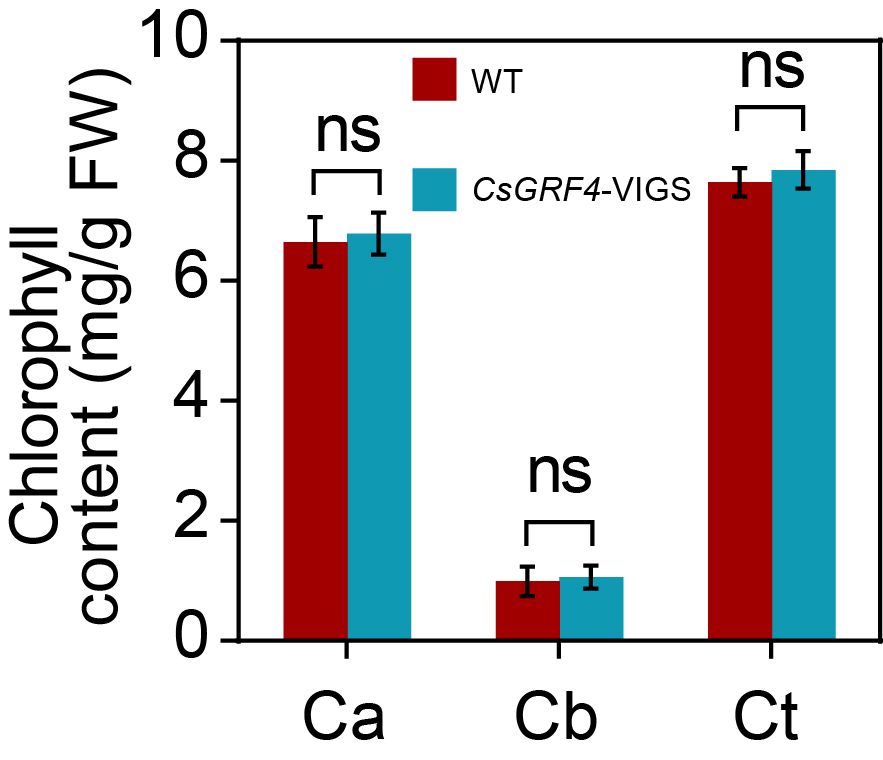


**Additional file 9: Fig. S3. The chlorophyll content of WT and *CsGRF04*-VIGS plants before treatments.**

ns: not significant.
