# Supplementary material for: Genome-wide identification of the GRF family in sweet orange (Citrus sinensis) and functional analysis of the CsGRF04 in response to multiple abiotic stresses
Source: BMC Genomics. 2024 Jan 6;25:37. doi: 10.1186/s12864-023-09952-8 (PMC10770916; doi:10.1186/s12864-023-09952-8)
Supplement: Supplementary file 10 — Additional file 10: Fig. S4. Phenotype of 1-month-old WT and CsGRF04-VIGS plants under normal condition (NC) (left panels), after cold treatment (8 h at -4 °C) (middle panels) and after 3 days of recovery (right panels). The scale bar indicates 1 cm. [file 12864_2023_9952_MOESM10_ESM.docx]

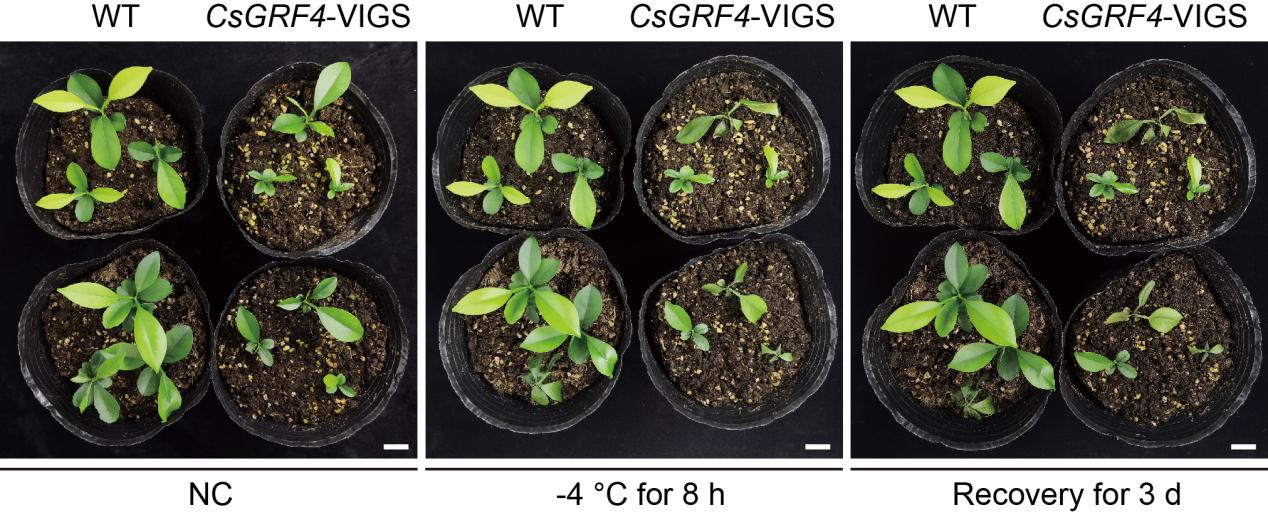


**Additional file 10: Fig. S4. Phenotype of 1-month-old WT and *CsGRF04*-VIGS plants under normal condition (NC) (left panels), after cold treatment (8 h at -4 °C) (middle panels) and after 3 days of recovery (****right panels).**

The scale bar indicates 1 cm.
